# Genome-wide discovery and differential regulation of conserved and novel microRNAs in chickpea via deep sequencing

**DOI:** 10.1093/jxb/eru333

**Published:** 2014-08-23

**Authors:** Mukesh Jain, VVS Narayana Chevala, Rohini Garg

**Affiliations:** Functional and Applied Genomics Laboratory, National Institute of Plant Genome Research (NIPGR), Aruna Asaf Ali Marg, New Delhi - 110067, India

**Keywords:** Chickpea (*Cicer arietinum*), deep sequencing, differential expression, microRNA, miRNA targets, tissue specificity.

## Abstract

MicroRNAs (miRNAs) are essential components of complex gene regulatory networks that orchestrate plant development. Although several genomic resources have been developed for the legume crop chickpea, miRNAs have not been discovered until now. For genome-wide discovery of miRNAs in chickpea (*Cicer arietinum*), we sequenced the small RNA content from seven major tissues/organs employing Illumina technology. About 154 million reads were generated, which represented more than 20 million distinct small RNA sequences. We identified a total of 440 conserved miRNAs in chickpea based on sequence similarity with known miRNAs in other plants. In addition, 178 novel miRNAs were identified using a miRDeep pipeline with plant-specific scoring. Some of the conserved and novel miRNAs with significant sequence similarity were grouped into families. The chickpea miRNAs targeted a wide range of mRNAs involved in diverse cellular processes, including transcriptional regulation (transcription factors), protein modification and turnover, signal transduction, and metabolism. Our analysis revealed several miRNAs with differential spatial expression. Many of the chickpea miRNAs were expressed in a tissue-specific manner. The conserved and differential expression of members of the same miRNA family in different tissues was also observed. Some of the same family members were predicted to target different chickpea mRNAs, which suggested the specificity and complexity of miRNA-mediated developmental regulation. This study, for the first time, reveals a comprehensive set of conserved and novel miRNAs along with their expression patterns and putative targets in chickpea, and provides a framework for understanding regulation of developmental processes in legumes.

## Highlight text

Deep sequencing led to the discovery of conserved and novel miRNAs and their targets, and highlighted the specificity and complexity of miRNA-mediated developmental regulation in chickpea.

## Introduction

Small non-coding RNAs control gene expression at transcriptional and post-transcriptional levels to regulate diverse developmental processes in eukaryotes (Bartel and Bartel, 2003; Carrington and Ambros, 2003; Finnegan and Matzke, 2003; Hunter and Poethig, 2003). MicroRNAs [miRNAs; 20–24 nucleotides (nt)] are the key elements of highly conserved RNA-mediated gene regulatory system in both plants and animals. In plants, the biogenesis of miRNAs includes transcription of miRNA genes into primary miRNAs (pri-miRNAs) containing a stem-loop structure, which is processed into a precursor (pre-miRNA) by DICER-LIKE (DCL) protein (Park *et al.*, 2002; Reinhart *et al.*, 2002, Kim, 2005; Rogers and Chen, 2013). The pre-miRNAs are further processed to generate a duplex miRNA, which after removal of complimentary sequence (miR*) generates mature miRNA (Bartel, 2004; Kim and Nam, 2006). The mature single-stranded miRNA is incorporated into an RNA-induced silencing complex (RISC) leading to degradation of mRNA targets or translational inhibition (Jones-Rhoades *et al.*, 2006).

Plant miRNAs perform crucial roles in diverse developmental processes, including leaf development, flowering, formation and maintenance of the shoot, floral and axillary meristems, establishment of organ polarity, vegetative to reproductive transition, and response to environmental stimuli (Jones-Rhoades *et al.*, 2006; Mallory and Vaucheret, 2006; Bartel, 2009; Chuck *et al.*, 2009; Rubio-Somoza and Weigel, 2011). The crucial roles of miRNAs in plant development have been exemplified by the dramatic and pleiotropic developmental defects in the *Arabidopsis* mutants *ago1*, *dcl1*, *hen1*, *hyl1* and *hst*, which have an impaired miRNA-signalling pathway (Bohmert *et al.*, 1998; Telfer and Poethig, 1998; Jacobsen, *et al.*, 1999; Lu and Fedoroff, 2000; Park *et al.*, 2002; Chuck and Connor, 2010). Several reports have demonstrated that miRNAs control gene expression at posttranscriptional levels by targeting transcripts for cleavage or translational repression (Bartel, 2004; Voinnet, 2009; Rogers and Chen, 2013).

Genome-wide identification of miRNAs has been reported in several plant species through computational and cloning approaches (Chen, 2004; Sunkar *et al.*, 2005; Li *et al.*, 2013). A total of 4467 miRNAs from 51 plant species have been registered in miRBase (release 18; Griffiths-Jones *et al.*, 2008). Of these, 1156 miRNAs have been reported from legumes. Previously, the identification of miRNAs was based on cloning and/or approaches using computational prediction, mostly in model plants. However, the advent of high-throughput sequencing technologies has catalysed the discovery of miRNAs in non-model plant species as well. The discovery of novel miRNAs in model plants has also been enriched via deep sequencing of small RNA transcriptomes (Jeong *et al.*, 2011; Breakfield *et al.*, 2012). Further, the availability of several bioinformatic tools for miRNA prediction has facilitated the discovery of miRNAs in various plant species (Wang *et al.*, 2009; Zhu *et al.*, 2010; Yang and Li, 2011). Due to rapid increase in discovery of plant small RNAs, specific criteria for annotation of plant miRNAs have also been proposed (Meyers *et al.*, 2008). Plant miRNAs are highly complimentary to the target mRNAs, which allow rapid identification of their putative targets with a high level of confidence (Rhoades *et al.*, 2002, Jones-Rhoades *et al.*, 2004). Using bioinformatic tools, many target genes involved in a wide diversity of functions have been identified in various plant species (Bartel, 2004; Schwab *et al.*, 2005; Axtell and Bowman, 2008).

Among legumes, most miRNA studies have been confined to the model plants soybean and *Medicago* (Lelandais-Briere *et al.*, 2009; Joshi *et al.*, 2010; Zhai *et al.*, 2011; Turner *et al.*, 2012; Mantri *et al.*, 2013). In addition to developmental processes, miRNAs have also been shown to regulate *Rhizobia*–legume symbioses (Simon *et al.*, 2009; Zhai *et al.*, 2011; de Luis *et al*., 2012; Mantri *et al.*, 2013). Chickpea (*Cicer arietinum*) is another important legume crop rich in human dietary protein. Recently, the draft genome sequence and transcriptome profiling in various tissues/organs in chickpea have been reported (Garg *et al.*, 2011; Jain *et al.*, 2013; Varshney *et al.*, 2013; Singh *et al.*, 2013). However, there has not been genome-wide discovery of regulatory small RNAs in chickpea. Being an important crop plant, it will be imperative to identify these regulatory small RNAs in order to study their role in regulation of gene expression.

In this study, we sequenced the small RNAs from seven tissues/organs in chickpea via deep sequencing. Our analysis identified 618 putative miRNAs, including 440 conserved and 178 novel candidate miRNAs. Many of the miRNAs showed spatial variation in expression, suggesting their diverse roles in chickpea development. We identified the putative targets of most of the miRNAs, which belonged to diverse biological processes. Our work will help understanding of the developmental regulation mediated by miRNAs in different tissues in chickpea and other legumes.

## Materials and methods

### Plant material and sequencing of small RNA libraries

Chickpea (*Cicer arietinum* L. genotype ICC4958) plants were grown either in a culture room or in the field. Root and shoot tissues were collected from the 15-day-old seedlings grown in the culture room. Mature leaves, stems, flower buds, flowers, and young pods were collected from mature plants grown in the field. At least three independent biological replicates of all the tissues were harvested. Total RNA from the tissue samples was isolated using TRI reagent (Sigma Life Science). The pooled total RNA from three biological replicates of each tissue was used for library preparation using a Small RNA Sample Preparation Kit according to the manufacturer’s instructions (Illumina Technologies). Each small RNA library was sequenced in individual lane for 36 cycles using Illumina Genome Analyser II. The sequence data was obtained in FASTQ files for further processing. The quality of data was assessed using NGS QC Toolkit v2.3 (Patel and Jain, 2012). Whole small RNA sequence data generated in this study have been deposited in the Gene Expression Omnibus database under the series accession number GSE51300.

### Data pre-processing

The sequence data was pre-processed using modified perl script provided in the miRTools software (http://centre.bioinformatics.zj.cn/mirtools). The quality control step included removal of low quality (>30% of bases with Phred score <20) reads and trimming of reads containing adapter/primer contamination and poly-A tail. After quality control, redundant reads were removed to retain only the unique reads, and the read count for each sequence was recorded. Only the reads with length 18–30 nt were retained for further analysis. Before processing the data for miRNA prediction, all the filtered unique reads from each sample were screened against annotated non-coding RNA sequences, including plant snoRNA (Plant SnoRNAbase v1.2; http://bioinf.scri.sari.ac.uk/cgi-bin/plant_snorna/home), tRNA (Genomic tRNA Database; http://gtrnadb.ucsc.edu/download.html), and rRNA (RFAM, v11.0). The remaining reads were screened against repeat sequences from RepBase (release 09-22-2012; http://www.girinst.org/server/RepBase/) and chloroplast sequence (Genbank accession number NC_011163) from chickpea. The reads which mapped onto these database sequences were discarded.

### miRNA identification

The miRBase database provides a searchable online repository for known miRNA sequences and their associated annotations. We used miRBase (release 18; Griffiths-Jones *et al.*, 2008) to identify conserved miRNAs in chickpea small RNA libraries. The filtered reads from each tissue were mapped onto the plant miRNAs from miRBase using Bowtie (v0.12.7) alignment tool (Li and Durbin, 2009). A maximum of two mismatches were allowed for the alignment. For the identification of novel miRNAs, the remaining reads were mapped onto the chickpea genome sequence using Bowtie and putative precursor sequences of an optimal size (250bp) were extracted for each aligned read (Yang *et al.*, 2011). The secondary structures for the extracted genomic sequences were predicted using RNAfold from the Vienna RNA software package (Hofacker, 2003). The predicted secondary structures along with the genome mapping information were processed using the plant-specific parameterized miRDeep-P core algorithm (Yang and Li, 2011) for miRNA prediction. miRDeep-P is a probabilistic-model-based miRNA discovery tool with well-adjusted parameters according to the features of plant miRNA-processing mechanisms (Meyers *et al.*, 2008; Thakur *et al.*, 2011). The scoring system in the miRDeep-P core algorithm includes nuclear conservation, consistency with Dicer processing, RANDfold *P*-value ≤0.05, presence of 3’-overhang, presence of star miRNA evidence, no bifurcations in the precursor sequence, minimum 14bp in the duplex, and not more than a six nucleotide difference between mature and star miRNA lengths (Meyers *et al.*, 2008). The accuracy and performance of miRDeep-P with its characteristic plant-specific scoring system and filtering criteria has been well demonstrated in previous studies (Jeong *et al.*, 2011; Yang and Li, 2011). The mature miRNA candidates were clustered into families based on their sequence similarity using CDHIT tools.

### Prediction of miRNA targets and functional annotation

We used the psRNATarget server (http://plantgrn.noble.org/psRNATarget/) with default parameters for prediction of putative targets of the chickpea miRNAs. The server predicts targets of miRNAs based on scoring for reverse complementarity and target-site accessibility by calculating the unpaired energy required to open secondary structures around small RNA target sites on mRNA (Dai and Zhao, 2011). A stringent penalty score of ≤2.5 (lower scores are better) was used for high specificity and low noise in target prediction. The chickpea genome annotation (Jain *et al.*, 2013) was used to find the putative function of the predicted targets. Conserved domains in the targets were identified by searching them in the PFAM database using the HMMscan program. The enriched GO terms were predicted using the BiNGO tool (v2.3) with *P*-value cut-off of ≤0.05. The KOG class of the putative targets was assigned using KOGnitor database available at NCBI.

### miRNA gene expression analysis

To determine the abundance of each miRNA in different tissue samples, the normalization factor for each tissue sample was calculated using DESeq (Anders and Huber, 2010) in the R statistical programming environment. Further, the read count of each miRNA in different tissue samples was normalized with normalization factor. To identify the miRNAs with tissue-preferential expression, the expression of individual miRNAs in each tissue was ranked using the method described earlier (Breakfield *et al.*, 2012). Heatmaps showing expression profiles were generated using MultiExperiment Viewer (v4.8). Hierarchical clustering was performed using the Euclidean distance matrix with complete linkage rule.

### Validation of miRNA expression

To validate the miRNA gene expression results, we performed quantitative reverse transcription-polymerase chain reaction (qRT-PCR) analysis. Firstly, RNA enriched for small RNAs was isolated from each tissue using a *mir*Vana^TM^ miRNA Isolation Kit (Life Technologies, USA) according to the manufacturer’s instructions. The quality and quantity of each isolated RNA sample was assessed using Bioanalyser 2100 (Agilent Technologies). We performed qRT-PCR analysis using a *mir*Vana^TM^ qRT-PCR miRNA Detection Kit (Life Technologies, USA) following the manufacturer’s protocol. Primer sets (miRNA-specific stem-loop RT and forward primers, and universal reverse primer) for the selected miRNAs were designed (Supplementary Table S1, at JXB online) according to recommended guidelines (Kramer, 2011). RT reactions were performed in a final reaction volume of 10 µl at 37ºC for 30min followed by 95ºC for 10min for each sample using 25–50ng of the RNA. Further, each PCR reaction was assembled in a final reaction volume of 10 µl and incubated at 95ºC for 3min followed by 40 cycles of 95ºC for 15 s and 60ºC for 30s. A no-template control (without cDNA) PCR reaction was also kept for each primer set. All the PCR reactions were performed using Applied Biosystems 7500 Real-Time PCR System (Applied Biosystems, USA). The expression of *U6* snRNA was used as an internal control to normalize for variance in the quantity of RNA and input cDNA. The specificity of each PCR reaction was determined by melting curve analysis. At least two independent biological replicates of each sample and three technical replicates of each biological replicate were analysed by qRT-PCR. The mean C_T_ value (from three technical replicates) of each miRNA was normalized to the mean C_T_ value (from three technical replicates) of *U6* for individual tissue samples. For each biological replicate, the relative expression level of each miRNA in different tissue samples was calculated using the standard delta delta C_T_ method. The average expression levels from two biological replicates and standard deviation were calculated for each tissue sample. The correlation between sequencing and qRT-PCR based expression analysis results was calculated using the R programming environment.

## Results and discussion

Although many studies have focused on miRNAs in various plant species, miRNAs and their target genes remain largely unknown in chickpea, one of the most important legume crops. This study was aimed at genome-wide discovery of miRNAs, their expression profiles, and possible regulatory implications in the development of various tissues/organs in chickpea.

### Small RNA sequencing

**Fig. 1.**
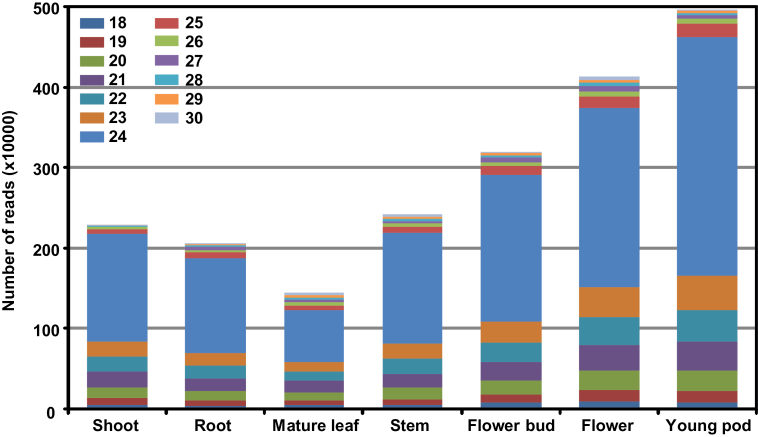
Size distribution of small RNA reads from seven chickpea tissues/organs. Number of high-quality filtered unique reads (after quality control and removal of redundant reads) of size 18–30 nt in different tissue samples is shown. This figure is available in colour at *JXB* online.

To perform genome-wide discovery of miRNAs in chickpea, we sequenced small RNA libraries constructed from shoots, roots, mature leaves, stems, flower buds, flowers, and young pod tissues of chickpea using the Illumina sequencing platform. A total of more than 154 million sequence reads were generated from all the tissues, in the range 17.7–28.2 million for individual tissue sample (Supplementary Table S2, at JXB online). After pre-processing (removal of low-quality reads, adapter/primer trimming, removal of duplicate reads, and size selection between 18 to 30 nt), the total number of distinct sequences were reduced to about 21 million. A total of 9.3% reads matched to structural non-coding RNAs (snoRNA, tRNA, and rRNA), repeat sequences and chickpea chloroplast genome sequence. After removal of these reads, the remaining 18 619 673 reads (small RNAs) were used for miRNA prediction. The number of unique small RNAs ranged from 1.2 to 4.6 million for individual tissues (Supplementary Table S2). The size distribution of the filtered sequence reads indicated the high-quality of the data (Fig. 1). The largest fraction (56%) of small RNAs were 24 nt long in all the tissues analysed, indicating the abundant representation of endogenous siRNAs. The 21-nt small RNAs accounted for 7.7%, 22 nt for 7.8%, and 23 nt for 8.4% of total small RNAs. Overall, more than 80% of the small RNAs were within the range of 21–24 nt, as expected. These observations were consistent with DCL cleavage products and those reported in previous studies (Jeong *et al.*, 2011; Breakfield *et al.*, 2012; Hwang *et al.*, 2013).

### Discovery of miRNAs

We employed two approaches for the discovery of miRNAs in chickpea. In the first approach, we identified conserved miRNAs based on similarity with plant miRNA sequences available in the miRBase database. A total of 15 460 unique reads from all the tissues mapped on the miRBase plant miRNAs, which resulted in the identification of a total of 302, 280, 248, 268, 247, 293, and 274 distinct conserved miRNAs from shoot, root, mature leaf, stem, flower bud, flower, and young pod, respectively (Supplementary Table S2). Many of these miRNAs were present in one or more tissues analysed. A comparative analysis of miRNAs identified from each tissue led to the identification of a total of 440 non-redundant distinct conserved miRNAs of size 20–24 nt in chickpea. The largest numbers of these miRNAs were conserved in legumes, soybean (118), and *Medicago* (80), followed by rice (70) (Supplementary Figure S1, at JXB online).

In the second approach, we performed *ab initio* prediction of novel miRNAs. The 18 604 213 distinct reads (excluding those mapped to miRBase) from all tissues were mapped to the chickpea genome (Jain *et al.*, 2013) individually from the seven tissues. About 70% of these reads mapped to the chickpea genome and were used for prediction of novel miRNAs using the miRDeep-P pipeline. The processing involved extraction of precursor sequences from the genome by extending mapped reads in the flanking regions, and its potential to form a stem-loop secondary structure. A total of 7 731 139 distinct potential precursor sequences were extracted and folded with the Vienna RNA package. From this pool, the typical miRNA precursor secondary structures were filtered and processed through the miRDeep-P pipeline. After processing, we identified a total of 109, 76, 123, 100, 106, 98, and 120 novel candidate miRNAs from shoot, root, mature leaf, stem, flower bud, flower, and young pod, respectively. Further, to generate a high-confidence set of novel miRNAs, we retained only those miRNAs represented by at least 10 reads in the tissue samples. This resulted in the identification of a total of 178 non-redundant novel miRNAs from all the tissues. All of these miRNAs displayed characteristics of genuine miRNAs (Meyers *et al.*, 2008). Interestingly, 47 of the 178 novel miRNAs showed significant similarity with conserved miRNAs, but were somewhat different. These miRNAs might represent newly evolved members of conserved miRNA families and were termed as novel miRNA isoforms. Some of these varied only in their sequence length, which might originate by differential cleavage of the pre-miRNA (Ebhardt *et al.*, 2010).

The minimum free energy (MFE) is commonly used as a measure of the stability of RNA secondary structure and has been used for miRNA prediction (Llave *et al.*, 2002; Reinhart *et al.*, 2002; Adai *et al.*, 2005; Zhang *et al.*, 2006). A lower MFE value signifies higher stability of RNA secondary structure and is a feature of miRNAs (Bonnet *et al.*, 2004). To analyse the accuracy of our prediction, we calculated the MFE of the predicted secondary structure of precursors for each miRNA in chickpea using the Vienna RNA package. We also calculated the MFE of known miRNAs from other plant species and animals. The analysis revealed the proper folding of chickpea miRNA precursors into characteristic stem-loop hairpin structures, as most (97%) of them showed MFE of less than –20 kcal mol^–1^. The chickpea miRNAs showed a broader distribution of MFE similar to other plants (Supplementary Figure S2, at JXB online). The overall distribution of MFE for chickpea miRNA precursors was very similar to that of plant miRNAs, but distinct from animal miRNAs (Supplementary Figure S2) as also reported previously (Thakur *et al.*, 2011). The average MFE for chickpea miRNA precursors was lower (–57.58 kcal mol^–1^), similar to that observed for other plant miRNA precursors (–56.83 kcal mol^–1^ in soybean, –67.73 kcal mol^–1^in *Medicago*, –76.2 kcal mol^–1^ in *Arabidopsis* and –71.57 kcal mol^–1^ in rice). To minimize the false predictions, the stability of secondary structures of all precursor sequences was statistically tested by enabling randomization tests using RANDfold. In addition, miRDeep implemented other criteria, such as seed conservation and presence of miRNA* evidence, as described in the materials and methods section, which ensured high-confidence prediction of miRNAs.

### Analysis of chickpea miRNAs

**Fig. 2.**
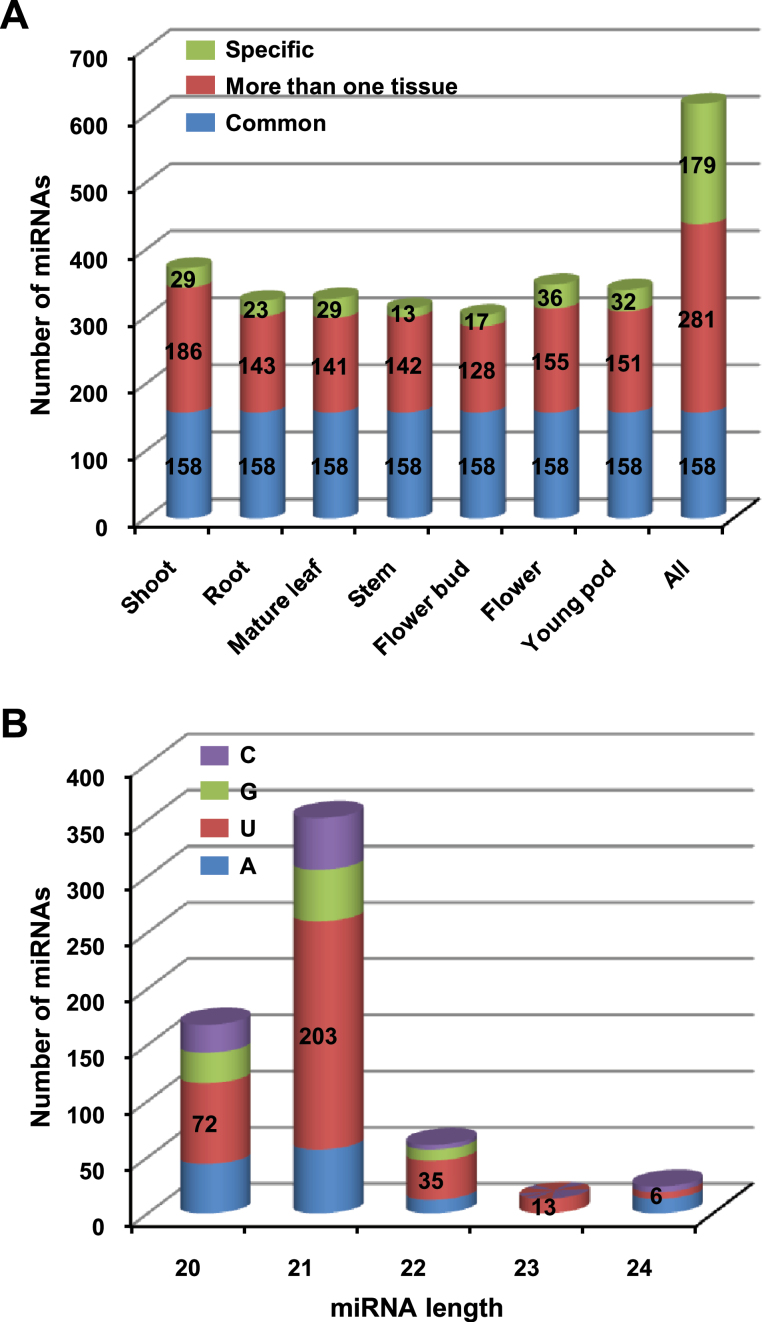
MicroRNA prediction in chickpea tissues. (A) Number of miRNAs identified in seven tissues are shown along with their specificity. The number of miRNAs identified in all the tissues (common), more than one tissue, and those specific to each tissue are given. (B) Size distribution of miRNAs and the identity of the first nucleotide. Most miRNAs are 21-nt long and have 5’-uridine. This figure is available in colour at *JXB* online.

Overall, we identified a total of 618 miRNAs from all the tissues. The total number of miRNAs from different tissues ranged from a minimum of 303 in flower buds to 373 in shoots (Fig. 2A; Supplementary Table S2). The largest numbers of novel miRNAs were identified from mature leaf (80), followed by shoot (71) and young pod (67). Of 618 miRNAs, 158 (26%) were present in all the tissues analysed and 281 (45%) were found in two or more tissues (Fig. 2A). A significant fraction (29%) of the miRNAs were identified only from a specific tissue sample (Fig. 2A).

The size distribution analysis showed the predominant (57%) representation of 21-nt-long miRNAs (Fig. 2B). About 27% of miRNAs were 20-nt-long, whereas only 16% were of 22–24 nt in length. Further, most miRNAs of different lengths harboured a uridine residue at the 5’-end. Similar size distribution and prevalence of 5’-uridine was observed for known miRNAs in other plant species (Supplementary Figure S3, at JXB online). The 21-nt-long miRNA with 5’-uridine is a characteristic feature of DCL1 cleavage and AGO1 association, which has been found in most known miRNAs (Reinhart *et al.*, 2002; Rajagopalan *et al.*, 2006; Mi *et al.*, 2008; Breakfield *et al.*, 2012). Further, the presence of multiple numbers of DCL and AGO proteins can produce miRNAs with different lengths, first nucleotide specificity and diverse functionality (Xie *et al.*, 2004; Rajagopalan *et al.*, 2006; Wu *et al.*, 2010; Cuperus *et al.*, 2011). For example, DCL3 produces 24-nt-long siRNAs and miRNAs (Wu *et al.*, 2010), and 24-nt miRNAs with 5’-adenine are a characteristic of AGO4 association (Mi *et al.*, 2008). It has also been reported that 24-nt miRNAs are recruited by AGO4 to induce methylation of their target genes (Wu *et al.*, 2010). The differences in miRNA size may make them functionally distinct. For example, 22-nt miRNAs can trigger the generation of secondary siRNAs from target mRNAs (Chen *et al.*, 2010; Cuperus *et al.*, 2010; Manavella *et al.*, 2012). In an earlier study, a larger number of 22-nt miRNAs were reported in legumes, *Medicago*, and soybean, than in any other plant (Zhai *et al.*, 2011).

We also analysed the nucleotide composition of mature miRNAs in chickpea and other plants. Most (76%) of the chickpea miRNAs had a GC content within the range of 31–60% (Supplementary Figure S4, at JXB online). The average GC content of mature miRNAs in chickpea (44%) was similar to that observed in other legumes (46% in soybean and 44% in *Medicago*) and *Arabidopsis* (44%), but less than that of *Populus* (50%), grapevine (50%) and maize (52%) (Supplementary Figure S4). It has been suggested that the GC content of miRNAs can be used as critical parameter for determining their biological roles (Mishra *et al.*, 2009). Altogether, the above results indicated the high-confidence prediction of miRNAs in chickpea. Detailed information about the identified chickpea miRNAs is given in Supplementary Table S3 (at JXB online).

### Identification of miRNA families

**Fig. 3.**
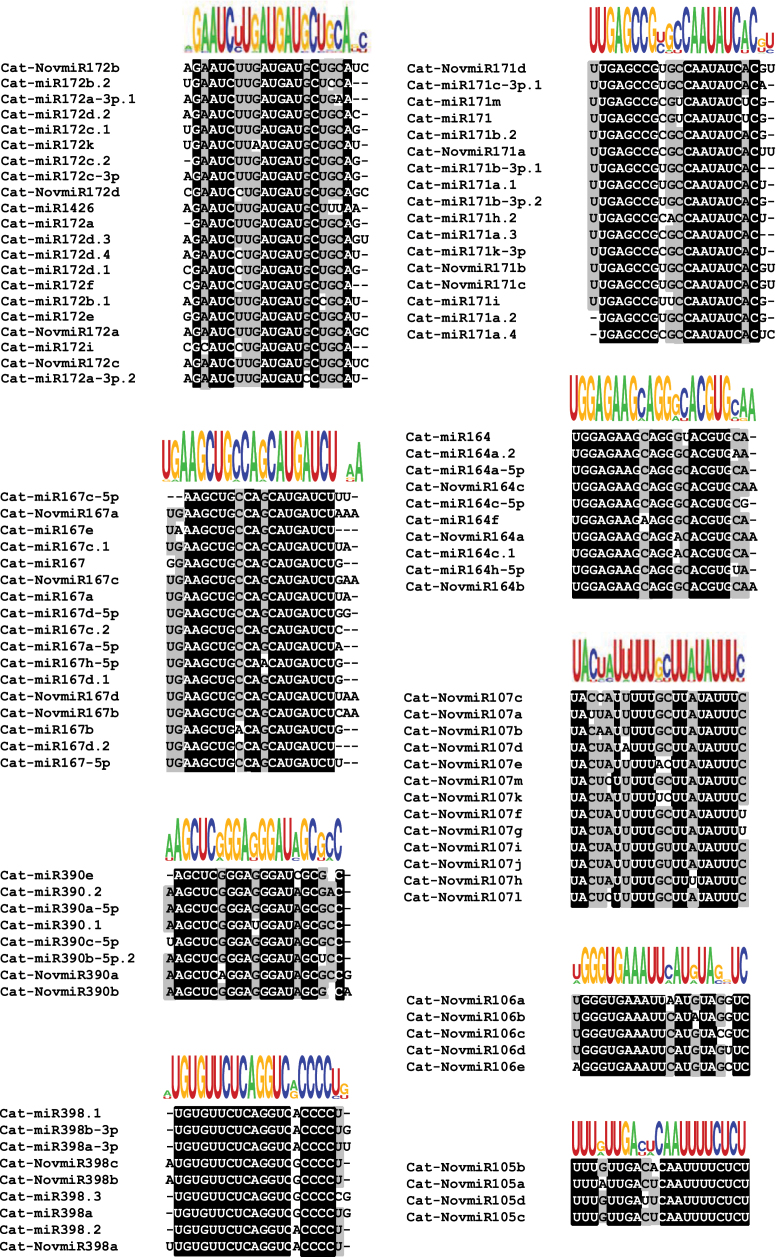
Clustering of conserved and novel miRNAs into families. Representative diagram showing the members of both conserved and novel miRNA families. The sequence alignment along with the consensus sequence logo of each family is shown. The nucleotides conserved in all the miRNAs of the same family are highlighted in black and those conserved in at least 50% of aligned sequences are highlighted in grey. This figure is available in colour at *JXB* online.

Based on sequence similarity, we clustered all the identified chickpea miRNAs into families. Of 618, a total of 197 miRNAs did not show significant similarity with any other miRNA, whereas the remaining 421 miRNAs were clustered into 73 families (Supplementary Figure S5, at JXB online). The number of miRNAs in each family varied from 2 to 24 (Supplementary Figure S5). Most of the families comprised 2–5 miRNAs; 28 families comprised at least five members. Such large gene families have also previously been reported in plants (Maher *et al.*, 2006; Li and Mao, 2007; Nozawa *et al.*, 2012). A large fraction (55.6%) of novel miRNAs represented unique sequences and were not clustered into families, which is consistent with their recent evolutionary emergence. However, some novel miRNAs clustered with conserved miRNA families (Fig. 3). At least eight families with a minimum two members comprised only novel miRNAs. A few representative examples of miRNA families are shown in Fig. 3. Many of the members within a family differ in their length and/or nucleotides at the 3’-end. The 21-nt members were most abundant in all family members, with few exceptions. The 22-nt variants were also significantly represented among various miRNA families. For example, five members each of the miR156 and miR172 families and four members each of miR164, miR167, and miR169 were of 22 nt in length. The 22 nt miRNAs have been shown to be necessary to trigger phasing of tasiRNA loci (Chen *et al.*, 2010; Cuperus *et al.*, 2010). The biological significance of mature miRNA length heterogeneity has been demonstrated in *Arabidopsis* (Vaucheret, 2009).

### Expansion and annotation of chickpea miRNAs

**Fig. 4.**
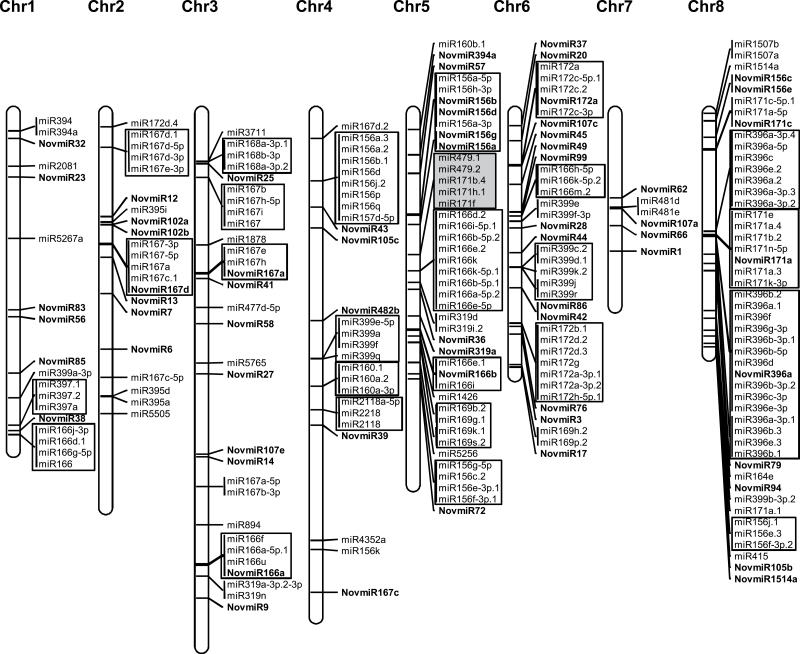
Distribution of miRNAs on eight chickpea chromosomes. The position of each miRNA has been marked. Novel miRNAs are distinguished in bold font. The miRNAs of the same family clustered together are shown in open boxes and miRNAs from different families clustered together in grey box.

To reveal the plausible reason for expansion of miRNA families in chickpea, we extracted the genomic coordinates of each miRNA and analysed their distribution in the chickpea genome. Of the 618 miRNAs for which genomic coordinates were available, only 228 (40%) could be located on eight linkage groups; the rest were present on scaffolds in the current version of chickpea genome assembly (Jain *et al.*, 2013). We observed substantial clustering (tandem arrays) of miRNAs on the eight linkage groups of chickpea (Fig. 4). Most of the clusters comprised the same miRNA family members. In some cases, the novel isoforms were also clustered with conserved miRNAs. More than one cluster of the same miRNA family was detected on the same or different chickpea chromosomes. For instance, two clusters of miR167 family members were found on each of chromosomes 2 and 3. A total of four clusters of miR156 family members were observed, one located on chromosome 4, two on chromosome 5, and one on chromosome 8. Four clusters of miR166, one on chromosome 1, two on chromosome 5, and one on chromosome 6, were detected. Two large clusters of miR369 family members were located on chromosome 8. Two members of miR479 and three members of miR171 families were clustered together on chromosome 5. Such tandem clustering of many miRNAs families has been observed in both *Arabidopsis* and rice (Jones-Rhoades and Bartel, 2004; Adai *et al.*, 2005; Cui *et al.*, 2009). Likewise, at least three miRNA families, miR166, miR399 and miR2601, were found organized into clusters in *Medicago* (Lelandais-Briere *et al.*, 2009). The expansion of plant miRNA gene families has been suggested to be mainly because of tandem and/or segmental duplication, which can cause dosage effects to regulate spatial and temporal gene expression in different species (Li and Mao, 2007; Cuperus *et al.*, 2011).

**Fig. 5.**
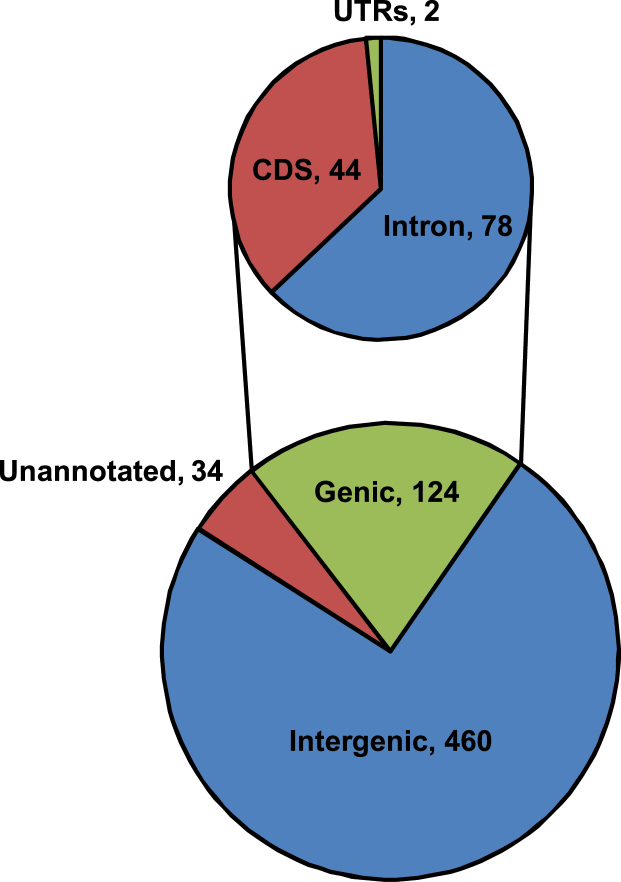
Annotation of 618 chickpea miRNAs. Distribution of miRNAs in different genomic features of the chickpea genome is shown in the Venn diagram. This figure is available in colour at *JXB* online.

To study the location of miRNAs in various genomic features, we integrated the genome annotation with the genomic coordinates of miRNAs. Consistent with the previous observations on plant miRNA genome localization (Kim, 2005), the majority (74.4%) of identified chickpea miRNAs were located in intergenic regions (Fig. 5). Interestingly, however, about 20% of miRNAs were of genic origin. Of the genic miRNAs, about 63% were located in the introns and 35.5% in coding regions, whereas only two miRNAs were located in untranslated regions (Fig. 5). A few earlier reports have also shown the origin of a considerable fraction of miRNAs from genic regions (Sunkar *et al.*, 2005; Brown *et al.*, 2008; Zhang *et al.*, 2013).

### Diversity of chickpea miRNA targets

To understand the biological functions of miRNAs, prediction of their messenger RNA (mRNA) targets is essential. We predicted the targets of all identified chickpea miRNAs using the computational algorithm psRNATarget, with stringent criteria. Of the 618 miRNAs, putative target mRNAs for 571 miRNAs (402 known and 169 novel) could be predicted (Supplementary Table S4, at JXB online). As about 80% of the gene space has been captured in the current version of the draft genome sequence of chickpea (Jain *et al.*, 2013), the target mRNAs for the remaining 124 miRNAs may not have been sequenced/annotated. It is also quite possible that these miRNAs do not have any targets or their targets could not be detected due to our stringent cut-off criteria. In total, 571 miRNAs targeted 1843 chickpea mRNAs. About 80% of miRNA targets were predicted to be regulated through cleavage and the rest by translational repression. This observation is in agreement with the earlier reports on plants, which showed mRNA cleavage as the predominant mechanism of miRNA-guided regulation (Schwab *et al.*, 2005; Rogers and Chen, 2013).

**Fig. 6.**
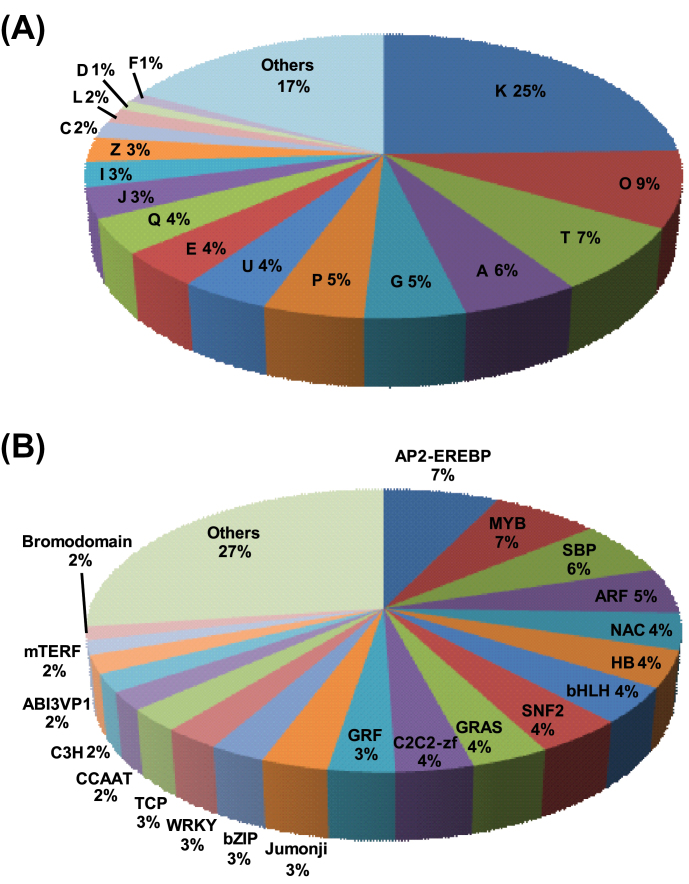
Functional annotation of predicted targets of chickpea miRNAs. (A) Pie chart showing distribution of different functional classes represented in the predicted targets. K, transcription; O, posttranslational modification, protein turnover, and chaperones; T, signal transduction mechanisms; A, RNA processing and modification; G, carbohydrate transport and metabolism; P, inorganic ion transport and metabolism; U, intracellular trafficking, secretion, and vesicular transport; E, amino acid transport and metabolism; Q, secondary metabolite biosynthesis, transport, and catabolism; J, translation, ribosomal structure, and biogenesis; I, lipid transport and metabolism; Z, cytoskeleton; C, energy production and conversion; L, replication, recombination, and repair; D, cell cycle control, cell division, and chromosome partitioning; F, nucleotide transport and metabolism. (B) Pie chart showing various transcription factor families represented in the predicted targets of chickpea miRNAs. This figure is available in colour at *JXB* online.

The number of predicted targets for miRNAs varied from one to as many as 20 (Supplementary Figure S6, at JXB online). The analysis of targets revealed the highest proportion (25%) of genes involved in the process of transcription (Fig. 6A; Supplementary Figure S7, at JXB online). In addition, we found that chickpea miRNAs may target transcripts encoding a wide range of proteins, such as those involved in post-translational modification and protein turnover (9%), signal transduction mechanisms (7%), RNA processing and modification (6%), carbohydrate, inorganic ion and amino acid transport (14%), and secondary metabolite biosynthesis (4%). GOSlim analysis also showed the highest abundance of biological process terms, regulation of transcription followed by oxidation-reduction, and protein phosphorylation (Supplementary Figure S7). The molecular function terms, ATP binding, DNA binding, and transcription factor activity, were most highly represented. In the cellular component category, nucleus was highest, followed by plasma membrane. Further, we analysed the prevalence of conserved PFAM domains in the targets (Supplementary Figure S8, at JXB online). Overall, the highest number of the predicted targets harboured a kinase domain, cytochrome P450, AP2 domain, MYB-domain, and RNA recognition motif, indicating the complexity of miRNA regulation.

Transcription factors encoding mRNAs constituted the major fraction of the miRNA targets. A total of 192 transcription factor-encoding mRNAs belonging to 51 families were predicted as targets of conserved miRNAs (Fig. 6B). Previous reports have also revealed transcription factors as the predominant targets of miRNAs (Bartel, 2004; Jeong *et al.*, 2011; Breakfield *et al.*, 2012; Hwang *et al.*, 2013). At least five members of 15 transcription factor families were predicted as targets of chickpea miRNAs. The members of AP2-EREBP (7%), MYB (7%), SBP (6%), and ARF (5%) were more often targeted by miRNAs (Fig. 6B). The largest numbers of miRNAs (39) were predicted to target MYB family transcription factors, followed by SBP (36), GRAS (33), and AP2-EREBP (30) families. Intriguingly, we found that 80% (12 of 15) of the total annotated SBP proteins in the chickpea genome were predicted as the targets of miRNAs (Supplementary Figure S9, at JXB online). Likewise, 48% (10 of 21) of ARF and 38% (6 of 16) of jumonji transcription factors were targeted by miRNAs. About 10% of the members of other transcription factor families were predicted as targets of chickpea miRNAs. As demonstrated earlier (Bartel, 2004; Kidner and Martienssen, 2005; Mallory and Vaucheret, 2006), the majority of transcription factor families targeted by conserved miRNAs were involved in plant development.

Novel miRNAs targeted genes encoding a broad range of proteins, including transcription factors (e.g. SBP, MYB, AP2-EREBP, HB, GRAS, and ARF), PPR proteins, F-box proteins, kinases, and defence-related proteins. On the other hand, several genes with unknown function were also predicted as the potential targets of chickpea miRNAs. In general, the predicted targets for novel miRNAs were more diverse than those of conserved miRNAs, an observation consistent with earlier reports (Jeong *et al.*, 2011; Hwang *et al.*, 2013).

### Conserved and novel targets of known chickpea miRNAs

Most conserved chickpea miRNAs targeted proteins with similar functions, as reported in other plants. For example, we found SBP transcription factors as targets of the miR156 family, ARF transcription factors for miR160, NAC for miR164, MYB for miR159, Scarecrow (GRAS) for miR171, HB for miR165/166, and AP2 for miR172 in chickpea (Supplementary Tables S4 and S5). The members of miR169, miR172, and miR2111 families targeted genes encoding F-box proteins harbouring diverse conserved domains, such as LRR, WD40, and kelch repeats. In addition to these families, several other miRNAs also targeted F-box protein-encoding genes. At least 11 novel miRNAs also targeted F-box protein-encoding genes. The F-box proteins are the components of proteasome-mediated degradation pathway and are involved in a variety of functions (Jones-Rhoades *et al.*, 2006; Lechner *et al.*, 2006; Ho *et al.*, 2008). Intriguingly, we identified some novel targets of a few conserved miRNA families as well. For example, in addition to SBP proteins, miR156 family members targeted homeobox and F-box proteins (Supplementary Table S5, at JXB online). A novel isoform of the miR156 family, NovmiR156f, was predicted to target an ARF protein. In *Lotus japonicus*, miR171c targeted a GRAS transcription factor, NSP2, required for root nodule symbiosis and miR397 targeted a laccase gene involved in copper homeostasis in the nodules (de Luis *et al*., 2012). We also found GRAS transcription factors and laccase protein encoding mRNAs as targets of chickpea miR171 and miR397 family members (Table 1; Supplementary Table S4). In addition, we found nodulation signalling pathway protein, putative cytochrome C biogenesis, aminocyclopropane-1-carboxylase, and kinesin-related protein encoding genes as targets of the miR171 family, which suggests another layer of complexity in these miRNA regulatory pathways. The miR398/299 targeted copper superoxide dismutase enzymes and miR395 targeted ATP sulfurylase, which are involved in protection against oxidative stress and inorganic sulphate assimilation, respectively (Kliebenstein *et al.*, 1998). The members of miR1507, miR2109, and miR2118 families targeted disease-resistance proteins similar to those in *Medicago* (Zhai *et al.*, 2011).

### Expression specificities of chickpea miRNAs

To gain insights into the putative roles of miRNAs, we analysed the expression profiles of all the 618 miRNAs in different tissues of chickpea. The expression (read count) of each miRNA in a tissue sample was normalized by a scaling factor calculated by DESeq (Anders and Huber, 2010). We observed significant variations in the expression profiles of chickpea miRNAs. Based on the normalized expression value, we classified the level of gene expression in five categories: very low, low, moderate, high, and very high (Supplementary Figure S10, at JXB online). The largest fraction (45–53%) of miRNAs showed a very low level of expression in all the tissues, followed by low expression (15–23%). A significant fraction (22–33%) of miRNAs were expressed at high to very high levels (Supplementary Figure S10). Further, we detected broad differential expression patterns for miRNAs. Patterns showing fluctuations in miRNA expression from ubiquitous to specific across different tissues were observed. The expression of novel miRNAs was more different between the tissues analysed as compared to the conserved miRNAs (Supplementary Figure S11, at JXB online). The ubiquitously expressed miRNAs with lowest variance across the tissues can be used as an internal control for qPCR normalization (Supplementary Figure S12, at JXB online).

**Fig. 7.**
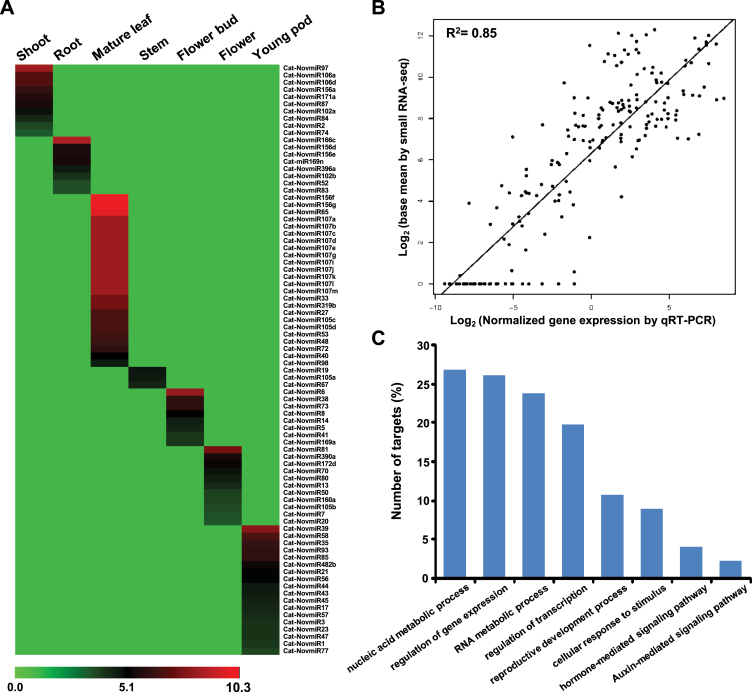
Expression analysis of chickpea miRNAs. (A) Heatmap showing tissue-specific expression of miRNAs. The scale represents log_2_ transformed normalized expression values. (B) Correlation of gene expression results obtained from small RNA-seq and qRT-PCR analysis for 28 selected miRNAs in seven tissue samples. A total of 196 data points (expression levels of 28 miRNAs in the seven tissue samples) are shown in the scatter plot. Each data point represent the log_2_ normalized expression level obtained from small RNA-seq (y axis) and qRT-PCR (x axis) analyses. (C) Significantly (*P*-value ≤0.05) enriched GO terms in the targets of chickpea miRNAs displaying tissue-specific expression. This figure is available in colour at *JXB* online.

For the identification of tissue-preferential and tissue-specific miRNAs, we excluded the miRNAs detected at very low level in all the tissues. The tissue preferential ranking was given to each miRNA as described (Breakfield, *et al.*, 2012). This analysis revealed preferential expression of several miRNAs in one or other tissue type(s) analysed. About 18.6% miRNAs were preferentially expressed in mature leaf and 16.8%, 14.7%, 14.4%, 13.1%, 12.3%, and 10% of miRNAs were preferentially expressed in young pod, flower, shoot, root, flower bud, and stem tissues, respectively (Supplementary Table S3). Further, we identified miRNAs exhibiting tissue-specific expression. Interestingly, at least 82 miRNAs displayed very high tissue specificity and all but one (miR169n) of these were novel miRNAs (Fig. 7A). The low number of tissue-specific members and non-representation of conserved miRNAs in this list might be due to the stringent criteria used for the analysis. The largest number of tissue-specific miRNAs was found in mature leaf followed by young pod as compared to other tissues (Fig. 7A). This implies that miRNA-mediated regulation of gene expression in leaf and pod tissues is more complex. Further, we detected several miRNAs with differential expression between flower bud and mature flower tissues, suggesting a tight control of flower development via miRNAs in chickpea. To validate the above miRNA expression analysis results, we performed qRT-PCR of at least 28 randomly selected miRNAs in all the tissue samples analysed. We observed a very good concordance in the expression patterns of miRNAs obtained by both the methods (small RNA-seq and qRT-PCR) as indicated by the overall correlation coefficient (0.85) (Fig. 7B). Many of these miRNAs exhibited significant differences (specific/preferential) in the expression levels across various tissues analysed (Supplementary Figure S13, at JXB online). Further, a comparative analysis showed similar (correlation >0.70) expression patterns of most (21 of 28) of these miRNAs obtained via small RNA-seq and qRT_PCR analyses (Supplementary Figure S14, at JXB online). Altogether, the spatio-temporal expression patterns revealed here will be very helpful in elucidation of specific biological roles of miRNAs in chickpea development.

Further, we also assessed the biological process GO terms significantly enriched in the targets of tissue-specific miRNAs. The genes involved in nucleic acid metabolic processes, regulation of gene expression, RNA metabolic pathway, reproductive development processes, and hormone-mediated signalling pathways were significantly enriched among these targets (Fig. 7C).

### Differential expression and target specificity of miRNAs in the same family

**Fig. 8.**
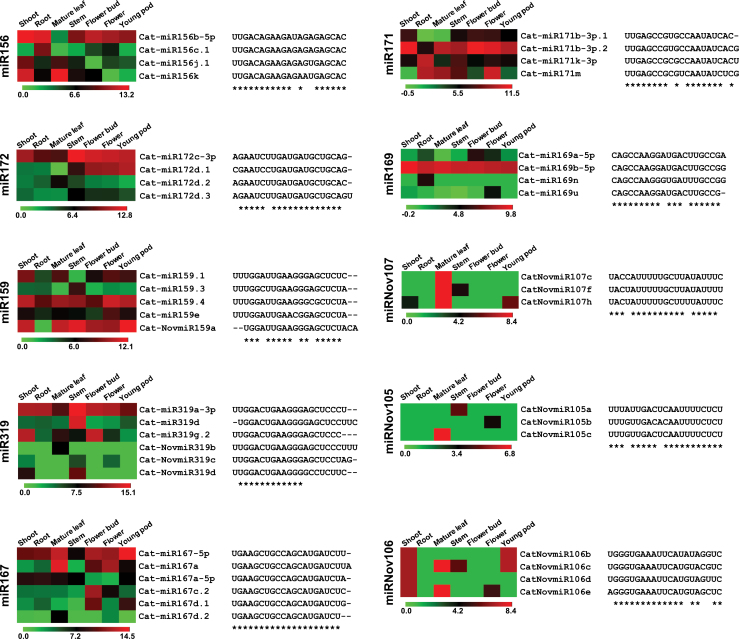
Heatmaps showing the differential expression of members of same miRNA family. The expression of miRNAs in different chickpea tissues is shown. Name of the miRNA family is indicated on the left side. The sequence of each miRNA is also shown on the right side. The colour scales represent log_2_-transformed normalized expression values.

The above analysis indicated that some miRNAs grouped into the same family did not always exhibit similar expression patterns. To gain more insights, we analysed the expression patterns of miRNAs families comprising more than two miRNAs. We observed several examples of miRNA families which exhibited differential expression across the tissues analysed. The differential expression of selected members of a few miRNA families, including miR156, miR159, miR166, miR167, miR171, miR172, and miR319, has been shown in Fig. 8 and Supplementary Figure S15 (at JXB online). For instance, miR156 was preferentially expressed only in mature leaf, whereas miR156b-5p was expressed in all the tissues except mature leaf. A higher expression of miR156c.1 was observed in root and flower tissues, and miR156k in tissues except flowers and young pods. The expression of miR156j.1 was limited to vegetative tissues only. Among the miR167 family members, miR167-5p was expressed in all the tissues, miR167a in mature leaf, flower tissues and young pod, miR167a-5p in all tissues except flower tissues, miR167c.2 only in flower tissues, miR167d.1 in flower bud and young pod, and miR167d.2 specifically in mature leaf. A novel isoform of the miR159 family, NovmiR159, was highly expressed in all the tissues except root, where its expression could not be detected. The members of novel miRNA families also exhibited differential expression patterns. For example, NovmiR107c was highly abundant in mature leaf, whereas NovmiR107f was expressed in mature leaf and stem, and NovmiR107h in shoot, mature leaf, and young pod. Three members of the miRNA family Nov105, NovmiR105a, b, and c, were specifically expressed in stem, flower, and mature leaf, respectively. Likewise, differences in the expression patterns of Nov106 miRNA family members were also observed. The differential expression of members of miRNA families (such as miR156, 159, 164, 166, 169, 319, 171, and 172) have also been reported in other plant species (Jeong *et al.*, 2011). It has been proposed that different isoforms of an miRNA can target distinct sets of mRNAs (Schwab *et al.*, 2005; Sieber *et al.*, 2007; Palatnik *et al.*, 2007), which can define complex specificities of spatio-temporal regulation. We also found the target specificity of miRNA isoforms of the same family in chickpea (Supplementary Table S4). For instance, the members of miR156 displaying differential expression targeted F-box protein (miR156b-5p and miR156c.1), 12-oxophytodienoate reductase (miR156j.1) and calcineurin-like metallo-phosphoesterase (miR156k) encoding genes. The miR159 family members targeted MYB transcription factors, aldehyde dehydrogenase, serine-threonine kinase protein, and a condensin complex subunit encoding genes. It has been argued that a single nucleotide difference in the sequence of miRNA family members can be sufficient to target a different set of mRNAs. However, these arguments remain to be substantiated. Overall, the diversity in expression patterns and mRNA targets can provide more functional significance to miRNAs in the regulation of developmental processes.

In conclusion, we present analyses of small RNA deep sequencing data sets generated from seven major tissues/organs of chickpea. Using these data, we identified a total of 618, including 440 conserved and 178 novel miRNAs, which targeted genes involved in diverse cellular processes. We detected differential expression of many conserved and novel miRNAs in different tissues/organs. We also showed the contrasting expression patterns of miRNA isoforms of the same family and their regulatory implications. Altogether, this study significantly extends the compendium of miRNAs and offers new perspectives for understanding the complex spatio-temporal regulation of transcription during organ development in chickpea and other legumes.

## Supplementary material

Supplementary data can be found at *JXB* online.

Supplementary Table S1. List of primer sequences used for qRT-PCR experiments.

Supplementary Table S2. Summary statistics of sequence data generated, quality control, and miRNA prediction.

Supplementary Table S3. Detailed description of all the miRNAs identified in chickpea.

Supplementary Table S4. Detailed information about the predicted targets of all the miRNAs.

Supplementary Table S5. Diversity of targets for miRNA families in chickpea.

Supplementary Figure S1. Number of miRNAs from other plant species conserved in chickpea.

Supplementary Figure S2. Frequency distribution of minimum free energy (MFE) of all miRNA precursors in chickpea, other plants and animals.

Supplementary Figure S3. Size distribution of miRNAs and frequency of 5’-nucleotide of miRNAs in chickpea and other plant species.

Supplementary Figure S4. Nucleotide composition of miRNAs in chickpea and other plants.

Supplementary Figure S5. Number of miRNA families of different sizes in chickpea.

Supplementary Figure S6. Number of miRNAs predicted with different number of targets.

Supplementary Figure S7. Most abundant (top 20) biological process, molecular function, and cellular component GOSlim terms represented in the predicted targets of chickpea miRNAs.

Supplementary Figure S8. Most abundant (top 20) PFAM domains represented in the predicted targets of chickpea miRNAs.

Supplementary Figure S9. Number of miRNAs targeting different transcription factor (TF) families and their frequency.

Supplementary Figure S10. Number of miRNAs with different expression abundances in various tissues.

Supplementary Figure S11. Heatmap showing expression profile of novel miRNAs in different tissues.

Supplementary Figure S12. Heatmap showing expression profile of ubiquitously expressed miRNAs.

Supplementary Figure S13. Quantitative reverse transcription polymerase chain reaction (qRT-PCR) analysis showing the relative expression levels of selected (28) miRNAs in different tissues of chickpea.

Supplementary Figure S14. Correlation between expression profiles of selected miRNAs obtained from small RNA-seq and qRT-PCR analysis.

Supplementary Figure S15. Heatmaps showing the differential expression of members of the same miRNA family.

## Funding

This work was financially supported by the core grant from NIPGR and the Department of Biotechnology, Government of India, under the Next Generation Challenge Programme on Chickpea Genomics (grant number BT/PR12919/AGR/02/676/2009 from 2009–2014). RG acknowledges the INSPIRE Faculty Award from the Department of Science and Technology, Government of India.

## Supplementary Material

Supplementary Data
