# Supplementary material for: Genome-wide discovery and differential regulation of conserved and novel microRNAs in chickpea via deep sequencing
Source: J Exp Bot. 2014 Aug 23;65(20):5945–58. doi: 10.1093/jxb/eru333 (PMC4203128; doi:10.1093/jxb/eru333)
Supplement: Supplementary Data [file supp_65_20_5945__index.html]

Genome-wide discovery and differential regulation of conserved and novel microRNAs in chickpea via deep sequencing — Genome-wide discovery and differential regulation of conserved and novel microRNAs in chickpea via deep sequencing — Supplementary Data 

# Genome-wide discovery and differential regulation of conserved and novel microRNAs in chickpea via deep sequencing

## Supplementary Data

Data files

**Files in this Data Supplement:**

- Supplementary Data - Supplementary Data
- Supplementary Data - Supplementary Data
- Supplementary Data - Supplementary Data
