# Supplementary material for: Genome-wide discovery and differential regulation of conserved and novel microRNAs in chickpea via deep sequencing
Source: J Exp Bot. 2014 Aug 23;65(20):5945–58. doi: 10.1093/jxb/eru333 (PMC4203128; doi:10.1093/jxb/eru333)

Supplementary data

Additional Supporting Information may be found in the online version of this article.

Table S1. List of primer sequences used for qRT-PCR experiments.

Table S2. Summary statistics of sequence data generated, quality control and miRNA prediction.

Table S3. Detailed description of all the miRNAs predicted in chickpea. (as separate excel file)

Table S4. Detailed information about the predicted targets of all the miRNAs. (as separate excel file)

Table S5. Diversity of targets for miRNA families in chickpea.

Figure S1. Number of miRNAs from other plant species conserved in chickpea.

Figure S2. Frequency distribution of minimum free energy (MFE) of all miRNA precursors in chickpea, other plants and animals.

Figure S3. Size distribution of miRNAs and frequency of 5' nucleotide of miRNAs in chickpea and other plant species.

Figure S4. Nucleotide composition of miRNAs in chickpea and other plants.

Figure S5. Number of miRNA families of different sizes predicted in chickpea.

Figure S6. Number of miRNAs predicted with different number of targets.

Figure S7. Most abundant (top 20) biological process, molecular function and cellular component GOSlim terms represented in the predicted targets of chickpea miRNAs.

Figure S8. Most abundant (top 20) PFAM domains represented in the predicted targets of chickpea miRNAs.

Figure S9. Number of miRNAs targeting different transcription factor (TF) families and their frequency.

Figure S10. Number of miRNAs with different expression abundances in various tissues.

Figure S11. Heatmap showing expression profile of novel miRNAs in different tissues.

Figure S12. Heatmap showing expression profile of ubiquitously expressed miRNAs.

Figure S13. Quantitative reverse transcription polymerase chain reaction (qRT-PCR) analysis showing the relative expression levels of selected (28) miRNAs in different tissues of chickpea.

Figure S14. Correlation between expression profiles of selected miRNAs obtained from small RNA-seq and qRT-PCR analysis.

Figure S15. Heatmaps showing the differential expression of members of same miRNA family.

Table S1. List of primer sequences used for qRT-PCR experiments.

miRNA ID	Stem-loop primer	Forward primer
Cat-miR2118a-5p	GTCGTATCCAGTGCAGGGTCCGAGGTATTCGCACTGGATACGACCTTTAC	GCAGGGATATGGGAGGGT
Cat-miR2199	GTCGTATCCAGTGCAGGGTCCGAGGTATTCGCACTGGATACGACGTGATC	GCAGTGATACACTAGCACGGA
Cat-miR5232	GTCGTATCCAGTGCAGGGTCCGAGGTATTCGCACTGGATACGACTCCAGG	CGCAGTACATGTCGCTCTCA
Cat-miR170-5p	GTCGTATCCAGTGCAGGGTCCGAGGTATTCGCACTGGATACGACTCTGAG	GCAGTATTGGCCTGGTTCA
Cat-miR319f	GTCGTATCCAGTGCAGGGTCCGAGGTATTCGCACTGGATACGACAAGAGG	GCAGTTGGACTGAAGGGG
Cat-miR393a-5p	GTCGTATCCAGTGCAGGGTCCGAGGTATTCGCACTGGATACGACGGATCA	CAGCAAAGGGATCGCAT
Cat-NovmiR6	GTCGTATCCAGTGCAGGGTCCGAGGTATTCGCACTGGATACGACGCAAGA	CGCAGTGAAAGACTGTGGA
Cat-NovmiR9	GTCGTATCCAGTGCAGGGTCCGAGGTATTCGCACTGGATACGACGTTCCC	CAGGCTTGGTGCAGGTC
Cat-NovmiR12	GTCGTATCCAGTGCAGGGTCCGAGGTATTCGCACTGGATACGACGAACAC	CGCAGGAGGAGTCTGACAT
Cat-NovmiR33	GTCGTATCCAGTGCAGGGTCCGAGGTATTCGCACTGGATACGACTGTAAT	CGCAGTTATCGGCAATGTTA
Cat-NovmiR64	GTCGTATCCAGTGCAGGGTCCGAGGTATTCGCACTGGATACGACCGGGTA	CGCAGATTCGGATGTGTAGA
Cat-NovmiR71	GTCGTATCCAGTGCAGGGTCCGAGGTATTCGCACTGGATACGACAACCTC	CGCAGAAAAAGTTCGAGGTT
Cat-NovmiR86	GTCGTATCCAGTGCAGGGTCCGAGGTATTCGCACTGGATACGACACCGTC	CGCAGCAATAGTTGTGATATGTT
Cat-NovmiR96	GTCGTATCCAGTGCAGGGTCCGAGGTATTCGCACTGGATACGACACCACT	AGAAGGGTCTGTTTGAGAGAAG
Cat-NovmiR101a	GTCGTATCCAGTGCAGGGTCCGAGGTATTCGCACTGGATACGACAGGATA	GCGCAGTTACTTCATCTTGTC
Cat-miR167c.2	GTCGTATCCAGTGCAGGGTCCGAGGTATTCGCACTGGATACGACGAGATC	CAGTGAAGCTGCCAGCAT
Cat-NovmiR66	GTCGTATCCAGTGCAGGGTCCGAGGTATTCGCACTGGATACGACTGAAGC	CGCAGATGGTATCAGGTCCT
Cat-NovmiR75	GTCGTATCCAGTGCAGGGTCCGAGGTATTCGCACTGGATACGACTCTTGT	CGCAGGAGTGAATCTTAGAACA
Cat-NovmiR103a	GTCGTATCCAGTGCAGGGTCCGAGGTATTCGCACTGGATACGACTTTGGG	GCCGTTTTTGTAGTGTATATCC
Cat-miR159d.1	GTCGTATCCAGTGCAGGGTCCGAGGTATTCGCACTGGATACGACGGGATC	GCAGAGCTGCTTAGCTATGG
Cat-miR167-3p	GTCGTATCCAGTGCAGGGTCCGAGGTATTCGCACTGGATACGACGGTGAA	GGACAGATCATGTGGCAGTT
Cat-miR166i-5p.2	GTCGTATCCAGTGCAGGGTCCGAGGTATTCGCACTGGATACGACGATCTT	GTCGAATGAGGTTTGATCCA
Cat-miR894	GTCGTATCCAGTGCAGGGTCCGAGGTATTCGCACTGGATACGACGGTGAA	GTTGCGTTTCACGTCAGGT
Cat-miR3711	GTCGTATCCAGTGCAGGGTCCGAGGTATTCGCACTGGATACGACAGGCCC	TGACTGGCGCTAGAAGGA
Cat-NovmiR18	GTCGTATCCAGTGCAGGGTCCGAGGTATTCGCACTGGATACGACACCATC	GCGCATTATACTTTGTTTGAT
Cat-NovmiR28	GTCGTATCCAGTGCAGGGTCCGAGGTATTCGCACTGGATACGACAAGCTA	GCGGTTTCCATTTTAGCTT
Cat-NovmiR79	GTCGTATCCAGTGCAGGGTCCGAGGTATTCGCACTGGATACGACATCTGC	GCAGCTAGAATTAGACATAAAAG
Cat-NovmiR88	GTCGTATCCAGTGCAGGGTCCGAGGTATTCGCACTGGATACGACCGTCTT	GCTGATGGATACGATGAAACT
OsU6	GTCGTATCCAGTGCAGGGTCCGAGGTATTCGCACTGGATACGACACCATT	GGGGACATCCGATAAAATTGG
Universal reverse primer	CCAGTGCAGGGTCCGAGGTA	

All primer sequences are from 5'to 3'direction.

Table S2. Summary statistics of sequence data generated, quality control analysis and miRNA prediction.

	Shoot	Root	Mature leaf	Stem	Flower bud	Flower	Young pod	Total
Raw reads	19183321	19775692	23004509	20063608	17701620	26642642	28233404	154604796
Non-redundant reads	2294215	2068961	1450608	2422490	3198310	4130124	4965404	20530112
Reads removed	229510	158871	270748	222951	295735	372529	360095	1910439
Reads used for miRNA prediction	2064705 (90.0%)	1910090 (92.32%)	1179860 (81.34%)	2199539 (90.8%)	2902575 (90.75%)	3757595 (90.98%)	4605309 (92.75%)	18619673 (90.70%)
Reads mapped to miRBase	2963	2381	1855	2009	1426	2814	2012	15460
**Conserved miRNAs predicted**	**302**	**280**	**248**	**268**	**247**	**293**	**274**	**440**
Reads mapped to genome	1442341 (69.96%)	1331111 (69.78%)	833842 (70.78%)	1552315 (70.64%)	2034168 (70.12%)	2578937 (68.68%)	3184150 (69.17%)	12956864 (69.64%)
Precursor extracted	1016016	929795	1031182	767313	1179877	1328435	1491765	7744383
**Novel miRNAs predicted**	**71**	**44**	**80**	**45**	**56**	**56**	**67**	**178**
**Total miRNAs predicted**	**373**	**324**	**328**	**313**	**303**	**349**	**341**	**618**

Table S5. Diversity of targets for miRNA families in chickpea.

miR family	Targets
miR156	Squamosa promoter-binding proteins, homeobox, ZF-HD and ABI3VP1 family transcription factors (TFs), F-box protein, SET domain protein, serine/threonine protein kinase, pentatricopeptide repeat (PPR)-containing protein, mitogen activated protein kinase kinase kinase, pectinesterase, metallophosphoesterase
miR157	Squamosa promoter-binding proteins, SET domain protein, DEAD-box ATP-dependent RNA helicase, PPR-containing protein, putative disease resistance protein
miR159	MYB and TCP family TFs, U-box containing protein, serine/threonine protein kinase, PPR-containing protein, alcohol dehydrogenase, subtilisin-like protease
miR160	ARF family TFs, laccase
miR164	NAC family TFs, glutathione S-transferase, F-box protein, E3 ubiquitin ligase, cell cycle regulated microtubule associated proteins
miR166	Homeobox, NAC, MYB and bZIP family TFs, serine/threonine protein kinase, PPR-containing protein, ribonuclease, cytochrome P450, F-box protein
miR167	Serine/threonine protein kinases, ARF and ZF family TFs, heat shock protein, fatty acyl-CoA reductase, receptor-like protein kinase, domain of unknown function
miR169	CCAAT, MYB and ABI3VP1 family TFs, F-box protein, calmodulin interacting protein, pectinesterase, transducin/WD40 repeat-like protein
miR171	GRAS family TFs, serine/threonine protein kinase, PPR-containing protein, phospholipid transporting ATPase, 14-3-3-like protein, globulin, vacuolar protein-sorting associated protein, cytochrome c biogenesis protein, kinesin-related protein, aminocyclopropane-1-carboxylase
miR172	AP2-EREBP family TFs, PPR-containing protein, serine/threonine protein kinase, vacuolar protein-sorting associated protein, F-box protein, L-ascorbate oxidase, ubiquitin conjugating enzyme, GDSL esterase
miR319	TCP and MYB family TFs, subtilisin-like protease, WD repeat-containing protein, beta-glucosidase, glucan endo-1,3-beta-glucosidase
miR390	Serine/threonine protein kinases, proline-rich receptor-like protein kinase, F-box protein
miR396	GRF family TFs, serine/threonine protein kinases, PPR-containing protein, receptor-like protein kinase, UDP-gycosyltransferase, splicing factor 3B subunit, nodulin family protein
miR397	Laccase, kinesin-like, pectinesterase, acylamino acid releasing enzyme
miR1426	AP2-EREBP family TFs, serine/threonine protein kinases, GRAS family TF, tetratricopeptide repeat protein
miR5208	Receptor-like protein kinase, COBRA-like protein, glucoronokinase, E3 ubiquitin ligase, disease resistance protein, dihyroorotase
miRNov13	F-box protein, serine/threonine protein kinase, exocyst complex component, formin-like protein, hypothetical protein
miRNov25	bZIP family TFs, zinc finger protein, SWI/SNF-SWI3 protein, peptidyl-prolyl cis-trans isomerase, unknown protein
miRNov37	Aux/IAA family protein, AP2-EREBP family protein, E3 uniquitin ligase, PPR-containing protein, polygalacturonase, galactose-1-phosphate uridyltransferase, unknown proteins
miRNov107	F-box protein, serine/threonine protein kinase, MYB, HLH, C_2C_2 GATA, homeobox, BTB/POZ and jumonji family TFs, cytochrome P450, , two component response regulator, PPR-containing protein, protein kinase, phosphodiesterase family protein, omega-3 fatty acid desaturase, cellulose synthase-like protein

Figure S1. Number of miRNAs from other plant species conserved in chickpea.

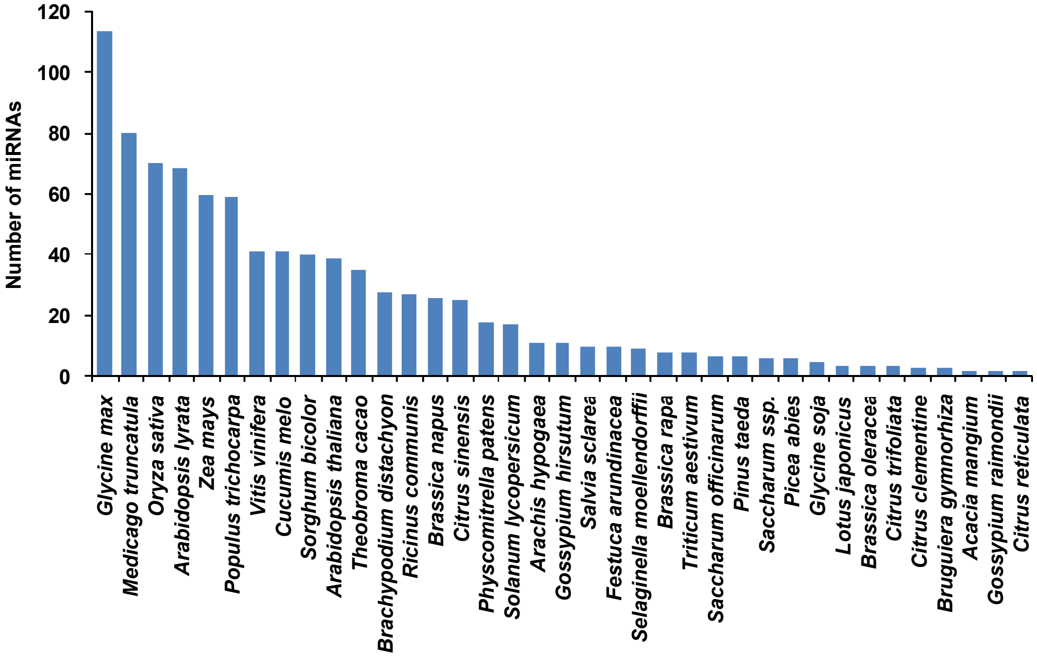

Figure S2. Frequency distribution of minimum free energy (MFE) of all miRNA precursors in chickpea, other plants and animals.

The precursors sequences of other species were extracted from the miRBase. The range of MFE given on x-axis represent negative values.

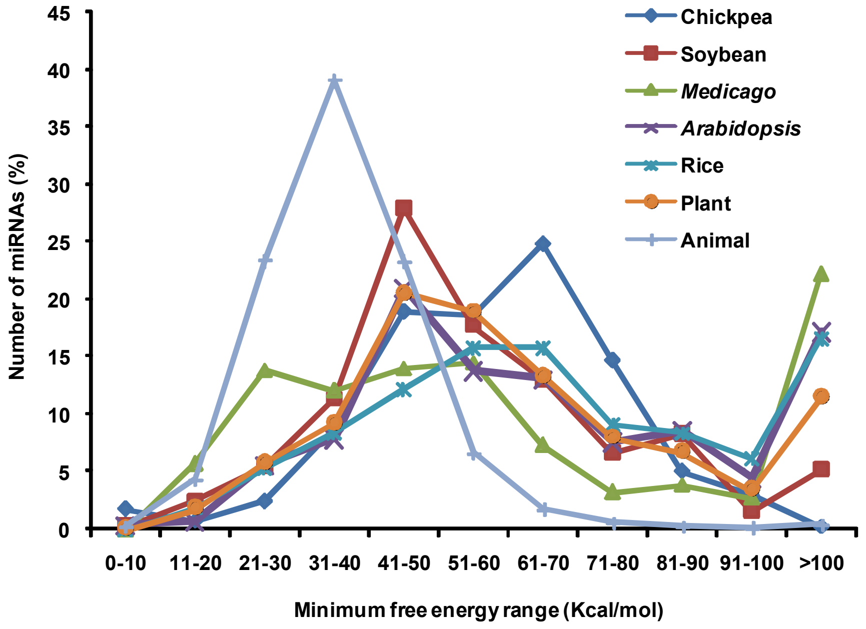

Figure S3. Size distribution of miRNAs and frequency of 5' nucleotide of miRNAs in chickpea and other plant species.

Higher abundance of 21 nt long miRNAs with uridine as a 5' terminal nucleotide in chickpea correlates with other species.

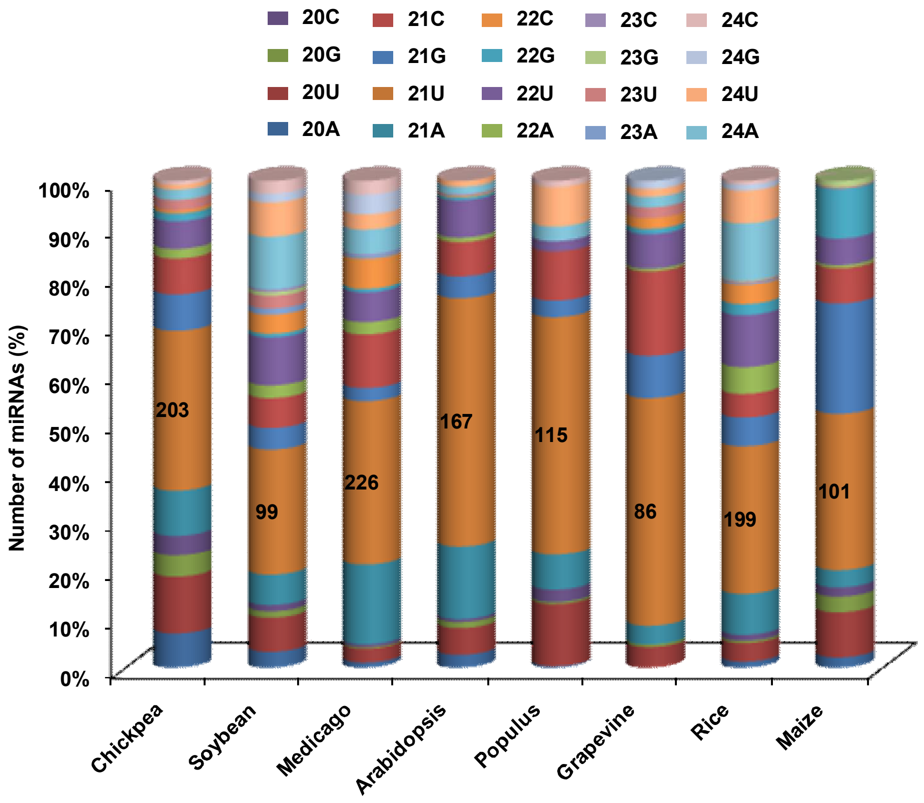

Figure S4. Nucleotide composition of miRNAs in chickpea and other plants.

(a) GC content distribution in mature miRNAs in chickpea. (b) Average GC and AT content in mature miRNAs from different plants.

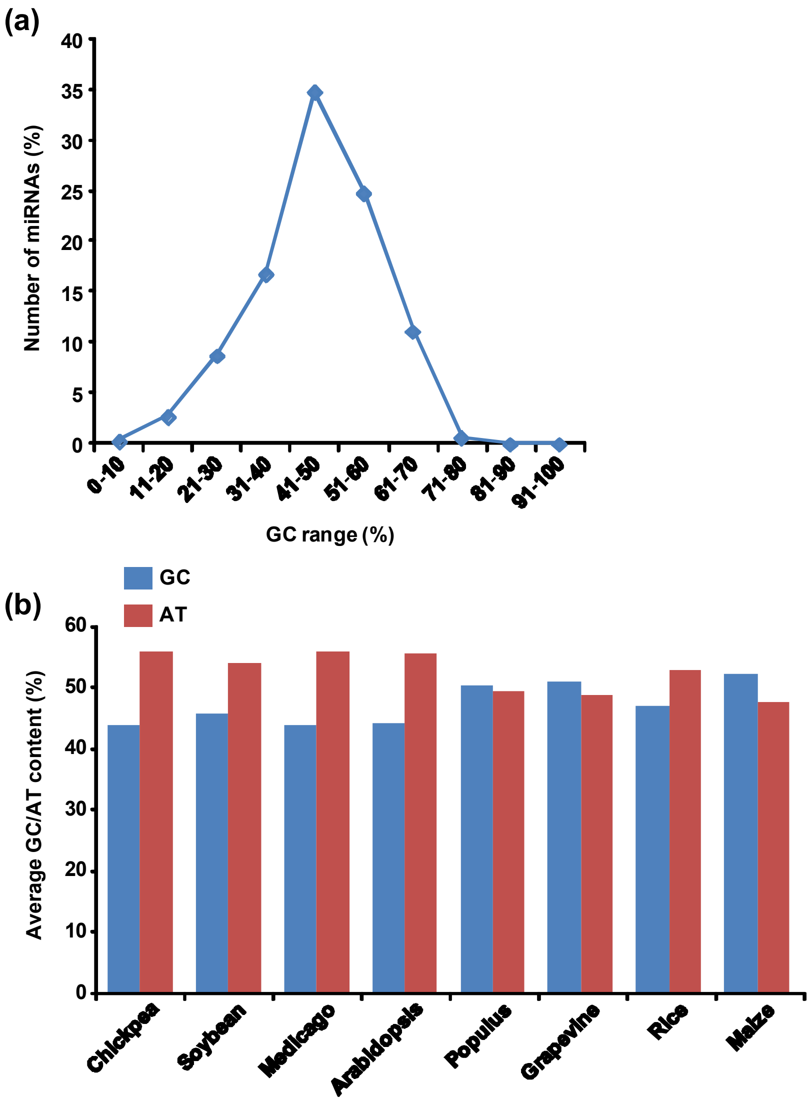

Figure S5. Number of miRNA families of different sizes predicted in chickpea.

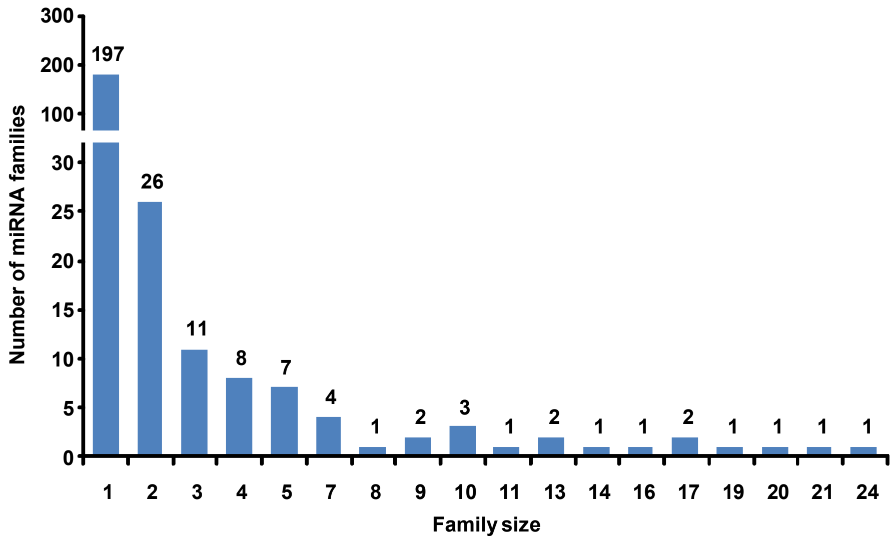

Figure S6. Number of miRNAs predicted with different number of targets.

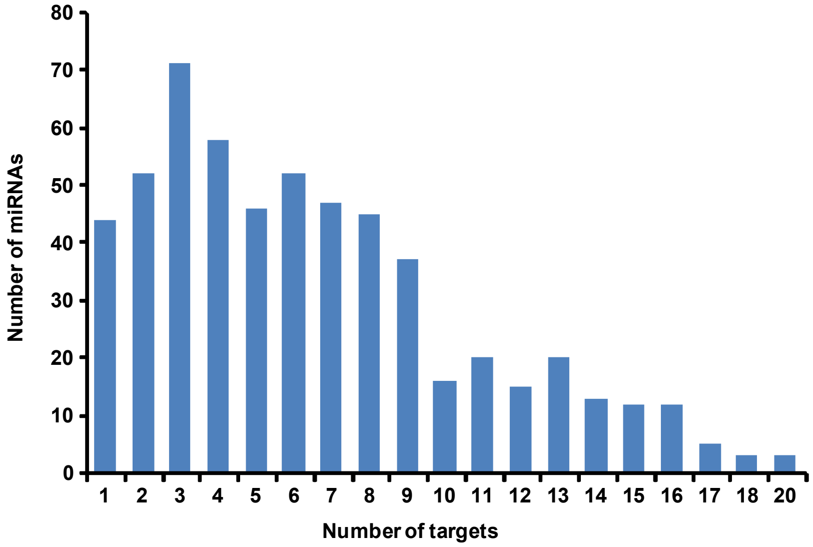

Figure S7. Most abundant (top 20) biological process, molecular function and cellular component GOSlim terms represented in the predicted targets of chickpea miRNAs.

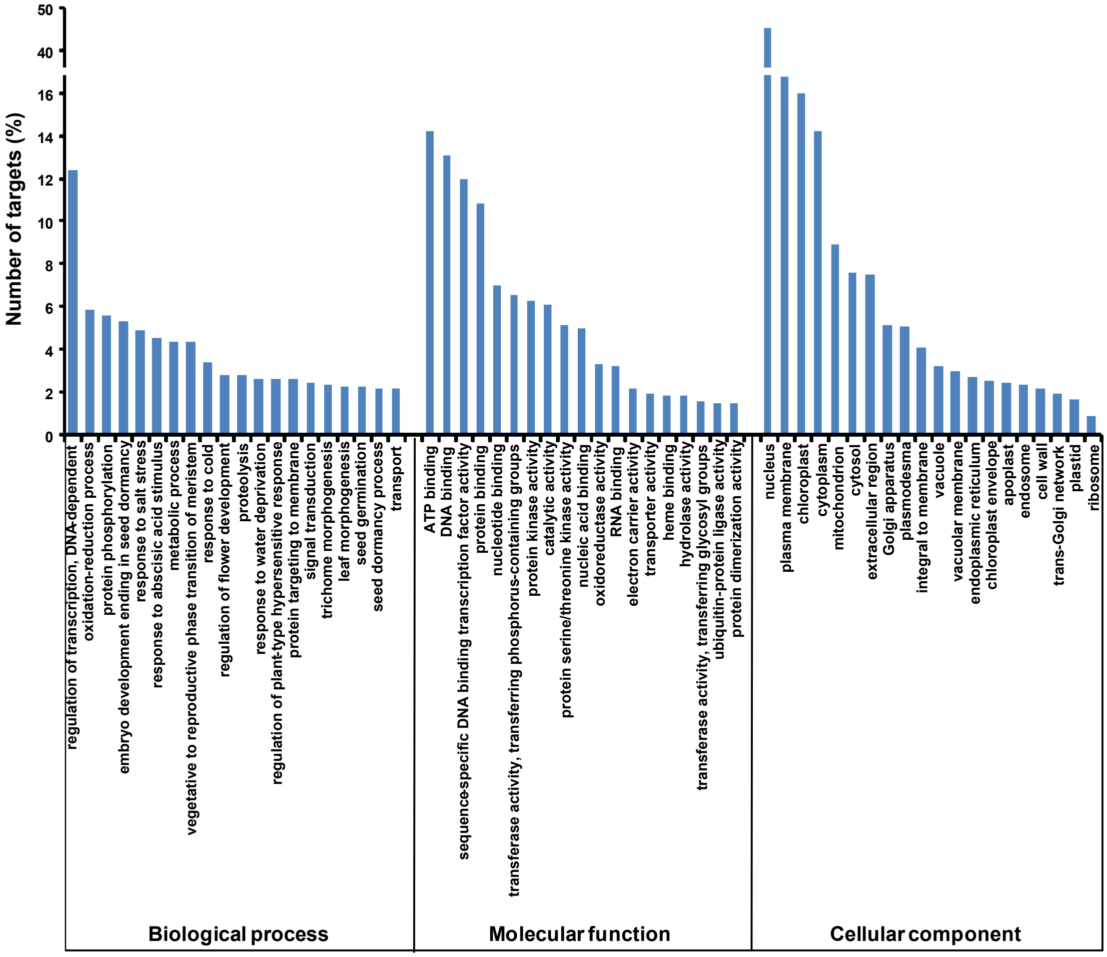

Figure S8. Most abundant (top 20) PFAM domains represented in the predicted targets of chickpea miRNAs.

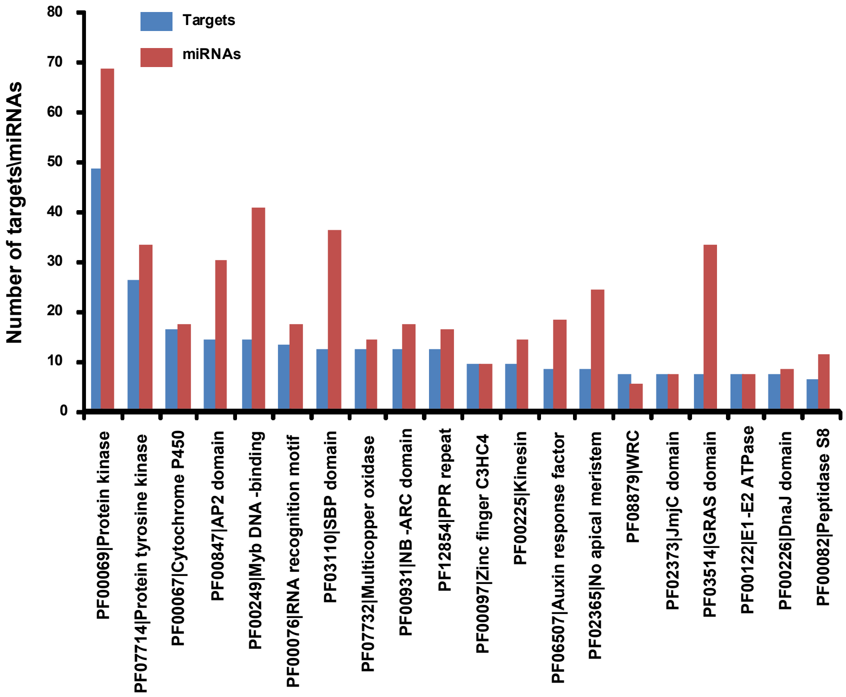

Figure S9. Number of miRNAs targeting different transcription factor (TF) families and their frequency.

The number of miRNAs (left y-axis) targeting different TF families (x-axis) are shown. Fraction of total TFs (right y-axis) belonging to different families, which are predicted as the targets of miRNAs are also shown.

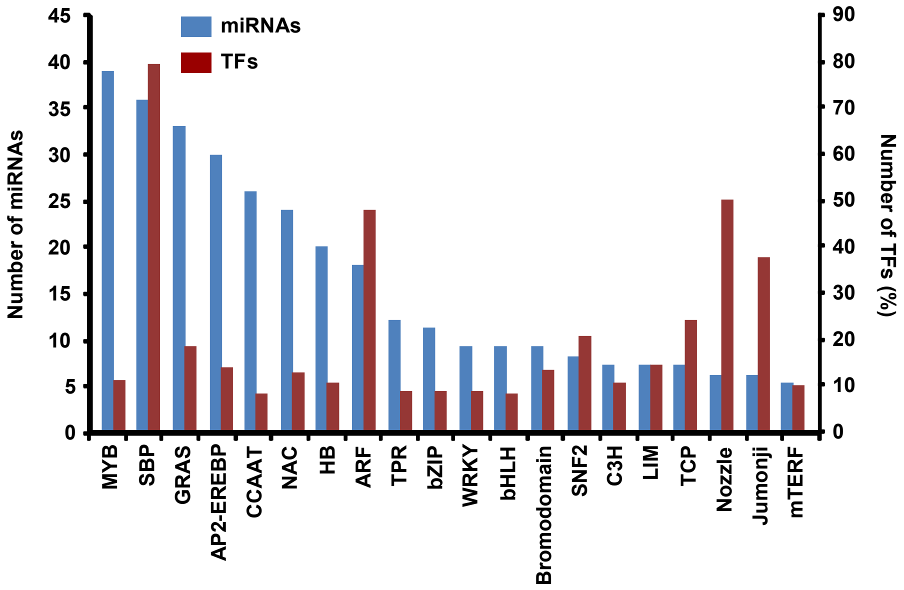

Figure S10. Number of miRNAs with different expression abundances in various tissues.

The miRNAs showing normalized expression values of Ò10, greater than 10 to 50, greater than 50 to 100, greater than 100 to 1000, and greater than 1000 have been classified as very lowly, lowly, moderately, highly, and very highly expressed, respectively.

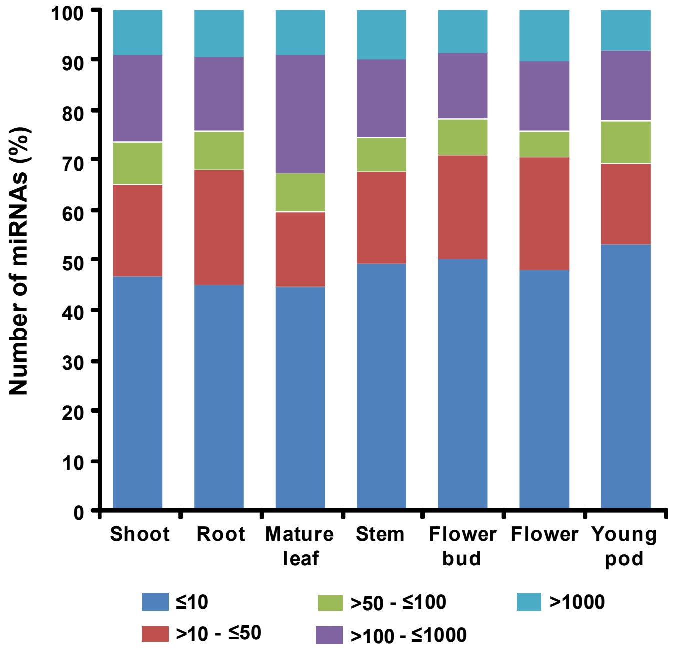

Figure S11. Heatmap showing expression profile of novel miRNAs in different tissues. The color scale represents $\log_2$ transformed normalized expression values.

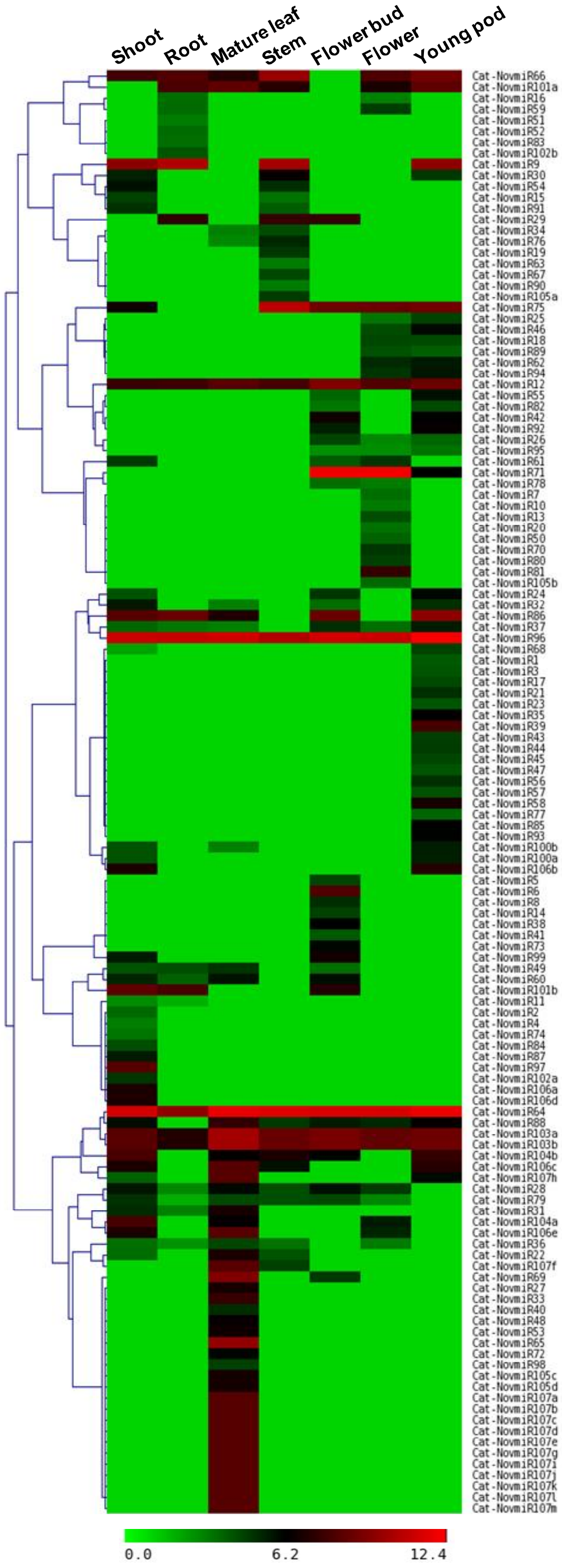

Figure S12. Heatmap showing expression profile of ubiquitously expressed miRNAs. The color scale represents log_2 transformed normalized expression values.

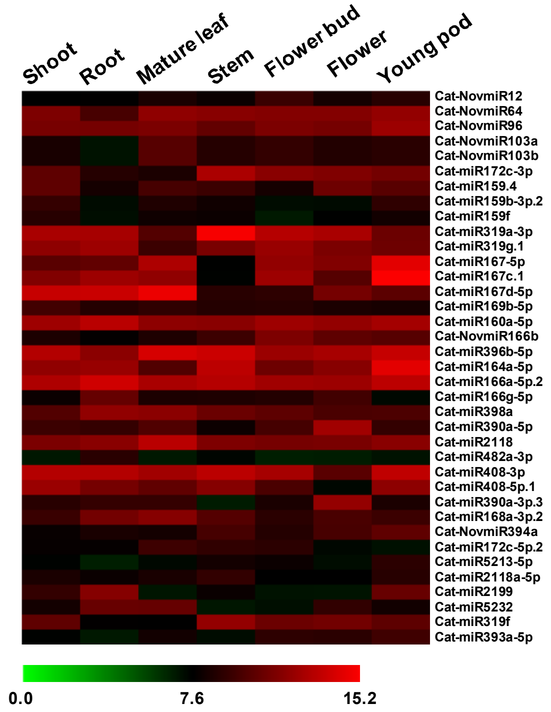

Figure S13. Quantitative reverse transcription polymerase chain reaction (qRT-PCR) analysis showing the relative expression levels of selected (28) miRNAs in different tissues of chickpea.

Histograms show relative expression levels of miRNAs in different chickpea tissues. The expression level of each miRNA in different tissues was normalized with expression of *U6* snRNA before calculation of relative expression levels among different tissue samples. The error bars indicate standard deviation among biological replicate tissue samples.

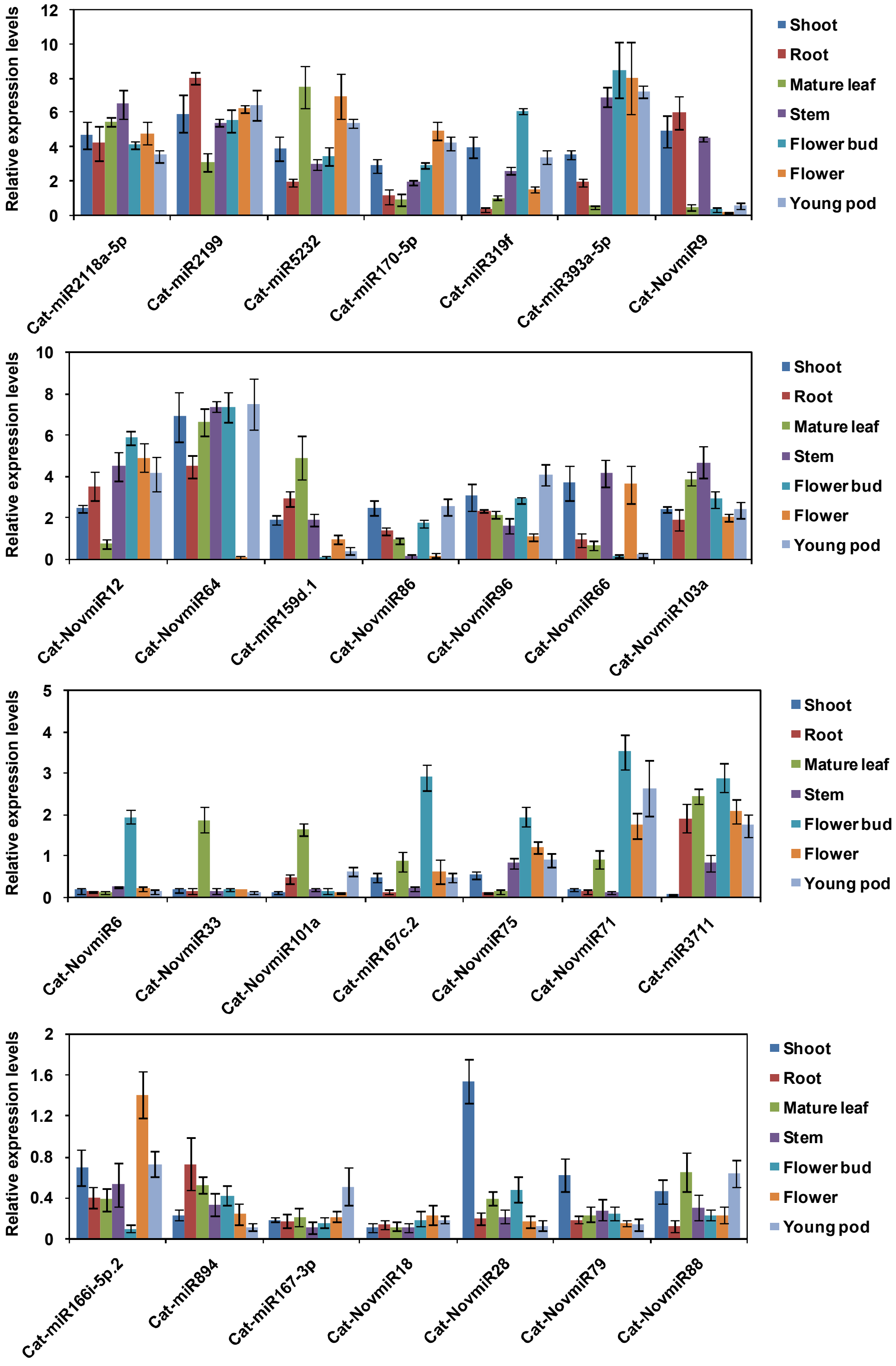

Figure S14. Correlation between expression profiles of selected miRNAs obtained from small RNA-seq and qRT-PCR analysis.

Heatmaps represent expression profiles of selected miRNAs (labeled on left side) obtained from small RNA-seq (left) and qRT-PCR (right) analysis. The color scales at the bottom represent log_2 transformed normalized expression values for small RNA-seq and qRT-PCR. The values between the two heatmaps represent correlation value between the expression profiles obtained from small RNA-seq and qRT-PCR analysis for each miRNA analyzed. The correlation values above 0.70 are highlighted in bold.

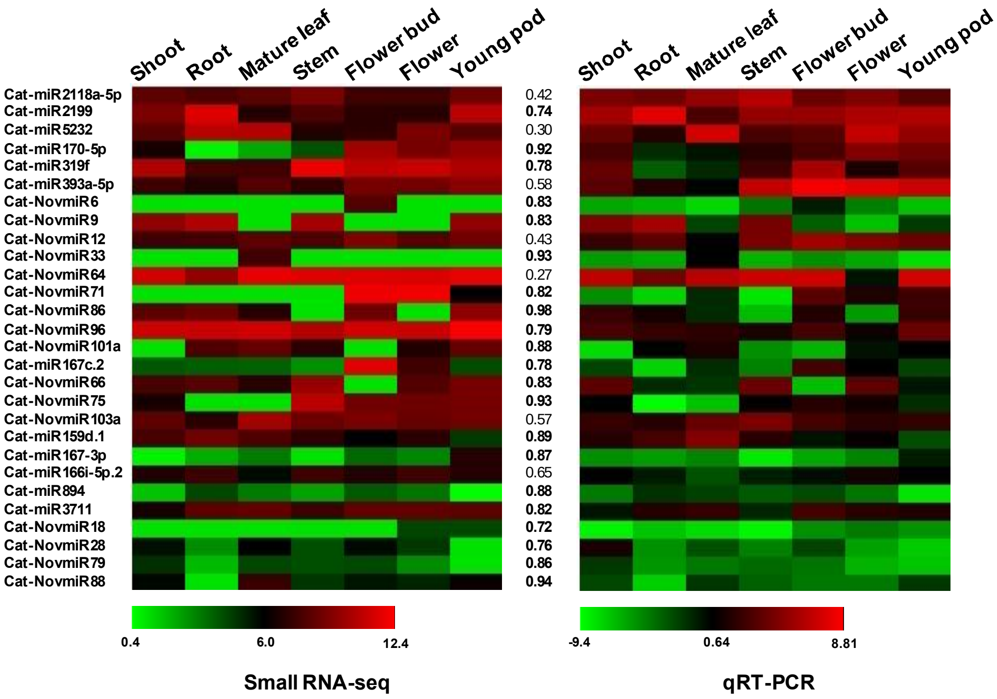

Figure S15. Heatmaps showing the differential expression of members of same miRNA family.

The expression of miRNAs in different chickpea tissues are shown. Name of the miRNA family is indicated on the left side. The sequence of each miRNA is also shown on the right side. The color scales represent log_2 transformed normalized expression values.

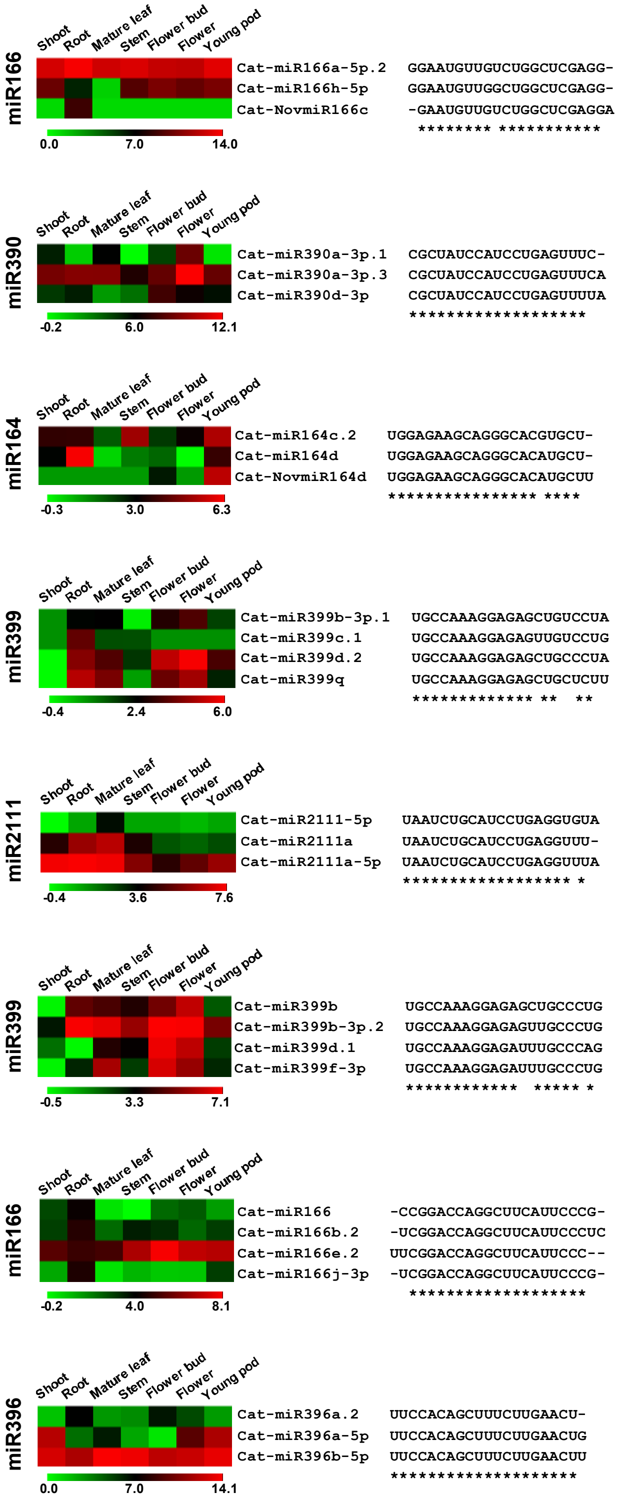

Supplement: Supplementary Data [file supp_eru333_jexbot127589_file001.pdf]
